# Histone deacetylase expression patterns in developing murine optic nerve

**DOI:** 10.1186/1471-213X-14-30

**Published:** 2014-07-09

**Authors:** Sarika Tiwari, Subramanian Dharmarajan, Mahesh Shivanna, Deborah C Otteson, Teri L Belecky-Adams

**Affiliations:** 1 Department of Biology, Center for Developmental and Regenerative Biology, Indiana University-Purdue University Indianapolis, 723 W Michigan St, Indianapolis IN-46202, India; 2 Center for Regenerative Biology and Medicine, Indiana University-Purdue University Indianapolis, 723 W Michigan St, Indianapolis IN-46202, India; 3 School of Optometry, MCPHS University, 10 Lincoln Sq., Worcester MA-01608, UK; 4 University of Houston College of Optometry, 4901 Calhoun Rd, Houston, TX 77204-2020, USA

**Keywords:** Histone deacetylases, HDACs, Mouse, Murine, Development, Optic nerve, Optic stalk, Astrocytes, Astrocyte precursors, Glial lamina

## Abstract

**Background:**

Histone deacetylases (HDACs) play important roles in glial cell development and in disease states within multiple regions of the central nervous system. However, little is known about HDAC expression or function within the optic nerve. As a first step in understanding the role of HDACs in optic nerve, this study examines the spatio-temporal expression patterns of methylated histone 3 (K9), acetylated histone 3 (K18), and HDACs 1–6 and 8–11 in the developing murine optic nerve head.

**Results:**

Using RT-qPCR, western blot and immunofluorescence, three stages were analyzed: embryonic day 16 (E16), when astrocyte precursors are found in the optic stalk, postnatal day 5 (P5), when immature astrocytes and oligodendrocytes are found throughout the optic nerve, and P30, when optic nerve astrocytes and oligodendrocytes are mature. Acetylated and methylated histone H3 immunoreactivity was co-localized in the nuclei of most SOX2 positive glia within the optic nerve head and adjacent optic nerve at all developmental stages. HDACs 1–11 were expressed in the optic nerve glial cells at all three stages of optic nerve development in the mouse, but showed temporal differences in overall levels and subcellular localization. HDACs 1 and 2 were predominantly nuclear throughout optic nerve development and glial cell maturation. HDACs 3, 5, 6, 8, and 11 were predominantly cytoplasmic, but showed nuclear localization in at least one stage of optic nerve development. HDACs 4, 9 and10 were predominantly cytoplasmic, with little to no nuclear expression at any time during the developmental stages examined.

**Conclusions:**

Our results showing that HDACs 1, 2, 3, 5, 6, 8, and 11 were each localized to the nuclei of SOX2 positive glia at some stages of optic nerve development and maturation and extend previous reports of HDAC expression in the aging optic nerve. These HDACs are candidates for further research to understand how chromatin remodeling through acetylation, deacetylation and methylation contributes to glial development as well as their injury response.

## Background

Astrocytes, once thought to be relatively inert cells, have been shown to play a multitude of roles in the developing, mature, and injured or diseased central nervous system [1,2]. For instance, astrocytes play key roles in synaptogenesis, maintenance of ion and transmitter levels, metabolic homeostasis, and support of neurons following injury [3]. Despite their importance, there still is not complete understanding of the factors necessary for the differentiation of these cells.

Within the mature optic nerve, there are 3 macroglial cell types: type I and type II astrocytes and oligodendrocytes [4]. Type I astrocytes arise from the PAX2-expressing neuroepithelial cells of embryonic optic stalk and a subpopulation of these cells migrate from the optic stalk onto the retinal surface where they associate with the surface vasculature to form the blood/retinal barrier [5-7]. Additional type I astrocytes from this lineage remain in the optic nerve adjacent to the globe within the glial lamina, a network of collagenous beams invested with astrocytes that is analogous to the lamina cribrosa in humans [8]. Oligodendrocytes, the myelin-forming cells of the CNS, arise from oligodendrocyte precursor cells that migrate from the ventral diencephalon into the optic nerve via the optic chiasm. Oligodendrocyte precursor cells also give rise to type II astrocytes, at least *in vitro,* but there are conflicting reports in the literature as to whether this also occurs *in vivo*[4,9]. There is increasing understanding of the secreted external and internal signals necessary for glial cells to develop, including leukemia inhibitory factor (LIF), bone morphogenetic proteins (BMPs), sonic hedgehog (SHH) and their intracellular signaling pathways [7,10-12]. There is also evidence that epigenetic factors cooperate with signaling in these three pathways [13,14]. However, little is known about the role that specific epigenetic factors play in the development of optic nerve glia.

Epigenetics is the study of factors that change transcriptional activity without changing the sequence of the DNA itself. These factors include DNA methylation and histone modifications [15]. Histones can be modified through post-translational modifications such as acetylation, phosphorylation, methylation, ubiquitination, and sumoylation [16]. Acetylation of histone tails is regulated by two large families of enzymes: histone acetyl transferases (HATs) and histone deacetylases (HDACs). Addition of acetyl groups to the histones offsets the positive charge on histone tails, resulting in relaxation of the chromatin structure, which, in turn, leads to greater accessibility of general and cell-type specific transcription factors to binding elements within the DNA [17]. HDACs are typically associated with transcriptional repression, although there are also reports that associate HDAC activity with transcriptional activation [18,19]. Further, both HATs and HDACs can respectively acetylate and deacetylate non-histone proteins in the cytoplasm, thereby affecting protein expression or function [20-25].

Whereas HDACs are known to play important roles in glial cell development and in disease states within other regions of the central nervous system, there is no information about HDAC expression patterns in developing optic nerve. Here, the spatio-temporal expression patterns of methylated histone 3 (K9), acetylated histone 3 (K18), and the HDACs 1–6 and 8–11 in the developing murine optic nerve have been determined, focusing on the peripapillary and glial laminar regions. Using RT-qPCR and western blot analysis, we established that HDACs 1–11 are expressed in optic nerve. Double-label immunohistochemistry indicated three patterns among optic nerve glia: 1) primarily nuclear localization (HDACs 1 and 2), 2) cytoplasmic and nuclear at one or more stages of development (HDACs 3, 5, 6, 8 and 11) and 3) cytoplasmic at all stages analyzed (HDACs 4, 9, 10). This study is a critical first step in identifying HDACs that may be involved in chromatin conformation during optic nerve astrocyte differentiation and will also establish a baseline for normal expression patterns of HDACs to enable comparison with disease states in the optic nerve.

## Results

### Acetylation and methylation in the murine optic nerve

The presence of acetylated (K18) and methylated (K9) histone 3, representing active and repressed chromatin within the genome respectively, was determined using triple-label immunofluorescence on sections through developing mouse optic nerve. Three different stages of development were chosen for these studies; embryonic day 16 (E16), which is a period when astrocyte precursors are found in the nascent optic nerve, postnatal day 5 (P5) when immature astrocytes are found throughout the optic nerve, including the optic nerve head and developing lamina and P30 when the glia within the lamina, as well as the optic nerve astrocytes and oligodendrocytes are mature [26-28].

**Figure 1:**
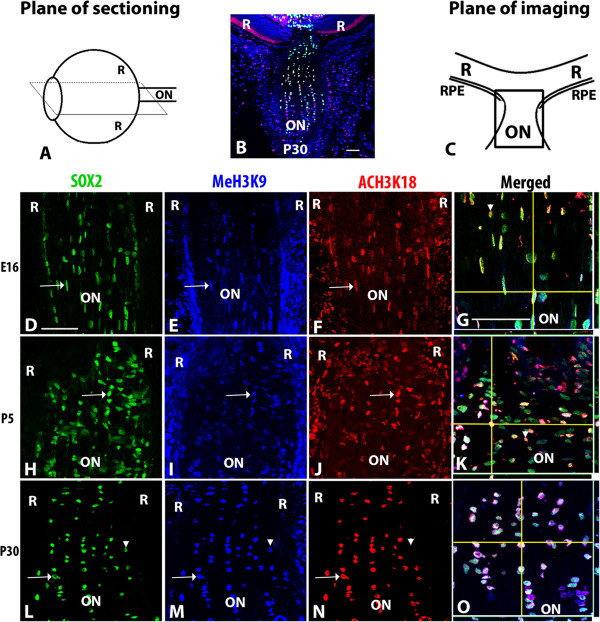
**Acetylated H3K18 and methylated H3K9 localization in the developing murine optic nerve. (A)** Drawing showing the plane of sectioning used throughout the study. **(B)** Low magnification view of the postnatal day 30 (P30) optic nerve (ON) optic nerve head and retina (R) stained with antibodies against SOX2 (green), MeH3K9 (blue) and acH3K18 (red). **(C)** Drawing showing the orientation of the optic nerve and retina in photomicrographs; the boxed area indicates the regions shown at higher magnification in panels **D-O**. Higher magnification of murine optic nerve is shown at E16 **(D-G)**, P5 **(H-K)** and P30 **(L-O)**. Arrows in **D-F**, **H-J** and **L-N** indicate triple labeled cells. Arrowheads indicate SOX2 positive cells co-labeled with MeH3K9 but negative for AcH3K18 **(G, K, L, M and N)**. Composite images in **G**, **K** and **O** show z stacks of optic nerve at high magnification; horizontal yellow lines correspond the x axis plane and vertical yellow lines corresponds to the y axis. The x, z axis is shown at the bottom of each panel, while the y,z axis is shown to the right of the panel. Abbreviations: R; retina, ON; optic nerve. Scale bars = 50 μm; Scale bar in **D** applies to **D-F**, **H-J**, and **I-N**, bar in **G** applies to **G**, **K** and **O**.

Sections through the optic nerve head and adjacent optic nerve were triple-labeled with antibodies against SOX2, which labels glia in the peripapillary, laminar and optic nerve regions, and antibodies against methylated histone H3 (MeH3K9) and acetylated histone H3 (AcH3K18) as markers of closed and open chromatin structure respectively. Within the optic nerve in the low magnification image of a triple-stained P30 section (Figure 1B), SOX2-immuno positive (SOX2+) glial cells located within 100 microns of the optic globe showed the characteristic elongation across the optic nerve that typifies the laminar region; glial nuclei located beyond the lamina were typically rounded or elongated in parallel to the optic nerve [29].

Within the optic nerve, the majority of SOX2+ nuclei were immunopositive for both MeH3K9 and AcH3K18. Along the external surface of the optic nerve, the majority of cells were SOX2 (-) and MeH3K9 (+). Within the population of extraocular SOX2 (-) cells, AcH3K18 staining was co-localized with MeH3K9 in a subset of cells. The SOX2(-), MeH3K9+ cells located furthest from the optic nerve head were most likely non-glial cells associated with the outer surface of the optic nerve that were revealed by the oblique angle of the section through the optic nerve.

At higher magnification, the majority of SOX2+ nuclei within the developing optic nerve at E16 (Figure 1D-G) and within the laminar area at P5 (Figure 1H-K) and P30 (Figure 1L-O) were co-labeled by antibodies for AcH3K18 and MeH3K9. The nuclei of many of the immature astrocytes at E16 were elongated in parallel to the optic nerve, consistent with the migration of astrocyte precursors towards the optic nerve head and retinal nerve fiber layer where they will differentiate into retinal astrocytes (Figure 1G). As development proceeded, nuclei of SOX2+ glial cells located in the optic nerve immediately posterior to the globe manifested the cross-wise elongation characteristic of the developing glial lamina (Figure 1K, O).

### mRNA expression levels in the murine optic nerve glia

**Figure 2:**
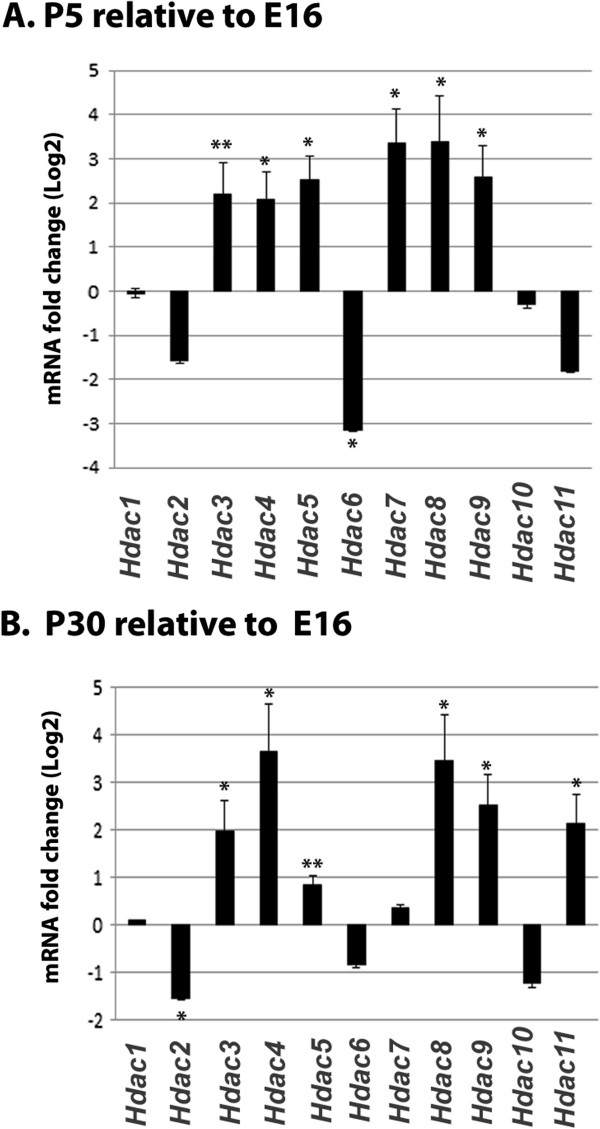
**mRNA expression levels in developing murine optic nerve.** RT-qPCR analysis of the relative levels of *Hdac* expression in E16, P5 and P30 murine optic nerve showed that *Hdac1-11* are expressed during all stages of glial cells development studied. **A** and **B** show the relative levels of expression for classical *Hdacs* in P5 and P30 murine optic nerve relative to the levels of E16 murine optic nerve. Bars show mean log(2) fold change ± SEM of three biological samples per timepoint. *, p = 0.001 vs. E16; **, p ≤ 0.005 vs. E16.

The presence of acetylated and methylated histones in optic nerve nuclei, coupled with published reports that glial cell fate decisions require deacetylation, led us to ask which HDACs might be involved in this process [30,31]. To determine which members of the large family of *Hdacs* were expressed in the optic nerve, reverse transcription quantitative polymerase chain reaction (RT-qPCR) was performed on murine optic nerve RNA. RT-qPCR clearly indicated that mRNAs encoding *Hdacs 1*–*11* were present at E16, P5, and P30 in the murine optic nerve (Figure 2). Each of the *Hdac* expression levels from E16, P5 and P30 were normalized to the geometric mean of β-2 microglobulin *(β2m),* signal recognition particle 14kDa (*Srp14*) and succinate dehydrogenase complex, subunit A (*Sdha*) and subsequently standardized to E16 levels for comparison (Figure 2A, B). Four expression level patterns emerged following analysis; 1) very little change in levels from E16 to P30 (*Hdac1*), 2) P5 and P30 levels that decreased relative to E16 (*Hdacs 2*, *6*, and *10*), P5 and P30 levels that increased relative to E16 (*Hdacs 3, 4, 5, 7, 8, 9*), and levels that decreased at P5 but increased at P30 (*Hdac11*).

### SOX2 positive cells in the developing murine optic nerve are astrocytes

**Figure 3:**
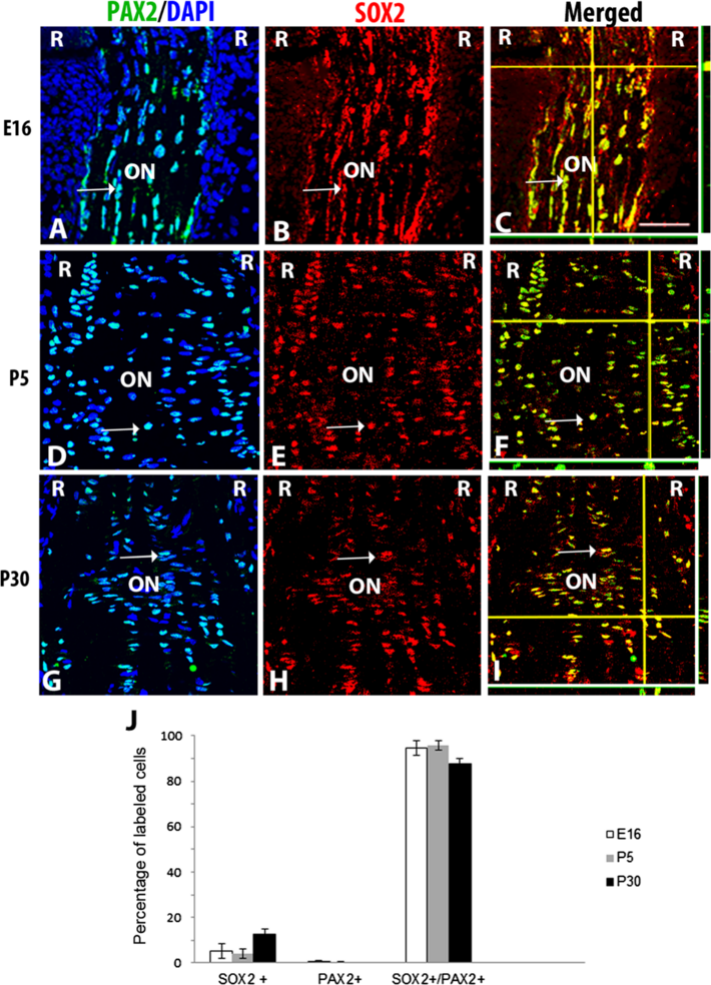
**PAX2 and SOX2 localization pattern in developing murine optic nerve*.*** Optic nerve sections at E16 **(A-C)**, P5 **(D-F)** and P30 **(G-I)** were triple-labeled with DAPI (blue), PAX2 (green) and SOX2 (red). An overlay of PAX2 and DAPI **(A, D, G)** is shown to aid in orientation of the images. Immunolabel of SOX2 and PAX2 (arrows) showed a majority of cells to be colabeled at all stages examined. Composite images in **C**, **F** and **I** show z stacks of optic nerve; horizontal yellow lines correspond the x axis plane and vertical yellow lines corresponds to the y axis. The x, z axis is shown at the bottom of each panel, while the y, z axis is shown to the right of the panel. Graph shown **(J)**, represents the percentage of cells positively labeled for SOX2 alone, PAX2 alone or SOX2 and PAX2 together in the proximal optic nerve. Bars show mean ± SEM of three samples per timepoint. Abbreviations: R; retina, ON; optic nerve. Scale bars = 50 μm. Scale bar in **C** applies to **A-I**.

**Figure 4:**
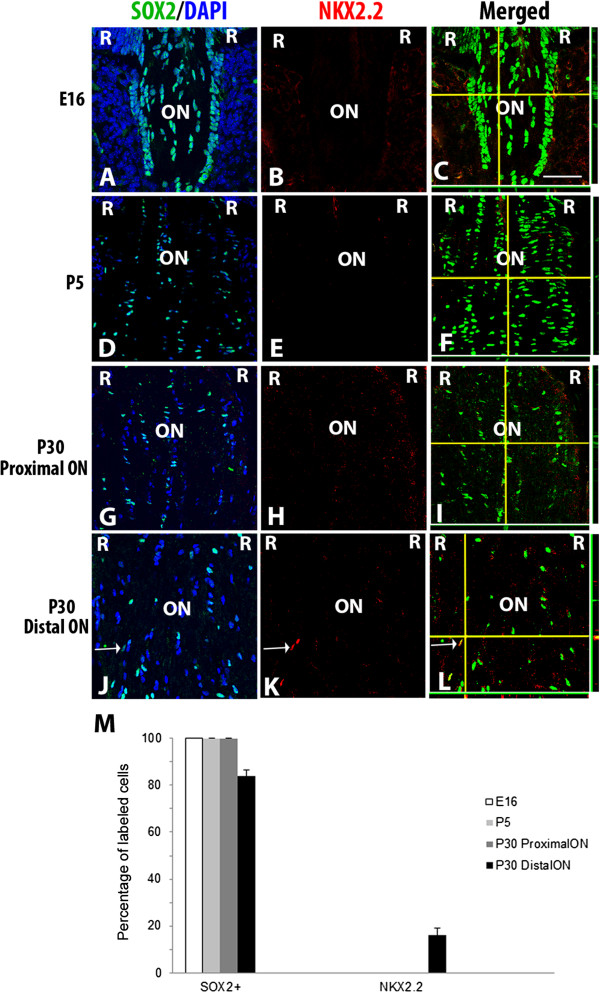
**NKX2.2 and SOX2 localization in developing murine optic nerve.** Optic nerve sections at E16 **(A-C)**, P5 **(D-F)**, P30 proximal to the eye **(G-I)** and P30 distal to the eye **(J-L)**, were triple-labeled with DAPI (blue), SOX2 (green) and NKX2.2 (red). An overlay of NKX2.2 and DAPI is shown to aid in orientation of the images **(A, D, G, J)**. At E16 and P5, NKX2.2+ cells were not detected in the proximal or distal optic nerve **(B, E)**. At P30, NKX2.2+ cells were not detected in the proximal nerve **(H)**, but were found in the distal optic nerve **(K)**. Composite images in **C**, **F**, **I** and **L** show z stacks of labeled optic nerve; horizontal yellow lines correspond the x axis plane and vertical yellow lines corresponds to the y axis. The x, z axis is shown at the bottom of each panel, while the y, z axis is shown to the right of the panel. Graph shown **(M)**, represents the percentage of cells positively labeled for SOX2 alone, NKX2.2 alone or both SOX2 and NKX2.2 together. Bars show mean ± SEM of three samples per timepoint. Abbreviations: R; retina, ON; optic nerve. Scale bars = 50 μm. Scale bar in **C** applies to **A-L**.

In order to investigate the nature of SOX2+ glial cells within the developing murine optic nerve, sections were double labeled using known markers for astrocytes (PAX2) (Figure 3) and oligodendrocytes (NKX2.2) (Figure 4) [32,33]. The majority of SOX2+ nuclei in the developing optic nerve at E16 (Figure 3A-C), P5 (Figure 3D-F), and P30 (Figure 3G-I) showed co-localization of SOX2 and the type I astrocyte marker, PAX2. Quantification of the cells at all three stages confirmed that a large majority of SOX2+ cells co-expressed PAX2; at E16 95% (±3%) were co-labeled, while 96% (±2%) and 88% (±2.5%) were co-labeled at P5 and P30 respectively (Figure 3J). Counts of the number of co-expressing cells in the proximal nerve (200μm from the exit of the nerve from the eye) at E16, P5 and P30 confirm the qualitative analysis of the sections (Figure 3J) and are consistent with observations that optic nerve astrocytes primarily populate the nerve at locations comparable to the lamina cribrosa in humans.

To examine the presence of oligodendrocyte precursors within the developing murine optic nerve, double-label immunohistochemistry was performed using antibodies that recognize SOX2 and oligodendrocyte precursor marker NKX2.2 [34]. For these experiments, we examined two regions for co-expression of SOX2 and NKX2.2 at P30: proximal optic nerve (up to 200μm from the exit of the nerve from the eye) and distal optic nerve (over 200μm from the optic nerve exit). At E16 (Figure 4A-C) and P5 (Figure 4D-F) no expression of NKX2.2 was detectable in the proximal or distal optic nerve. At P30, NKX2.2+ cells were not observed in the proximal optic nerve (Figure 4G-I) but were present in the distal optic nerve (Figure 4J-L). Quantification of the SOX2+ and PAX2+ cells revealed that 16.2% of the cells within the distal end of the optic nerve at P30 were NKX2.2+ and all of those cells showed co-localization with SOX2 (Figure 4M). These results are consistent with previous publications showing that oligodendrocyte precursors migrate into the rodent optic nerve postnatally [35].

### Patterns of Protein Localization in the developing optic nerve

**Figure 5:**
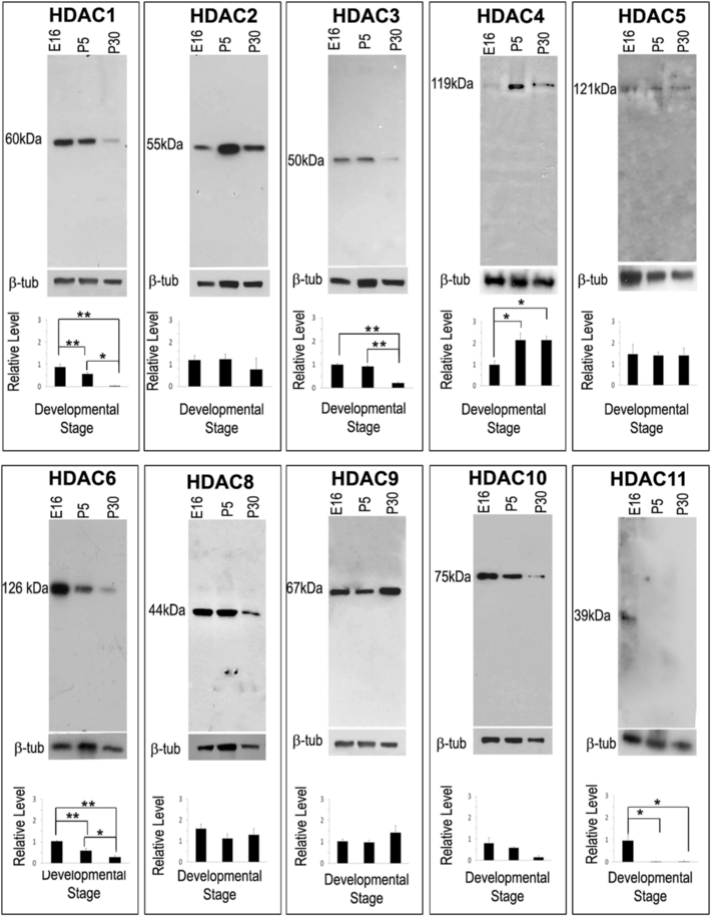
**Expression of HDACs in murine optic nerve.** Western blot analysis of HDAC proteins in E16, P5 and P30 murine optic nerve was performed to check the specificity of the antibodies used for the study. Blots were normalized to β-TUBULIN. Densitometric graphs for each HDAC panel show quantification of protein levels expressed as fold-change from E16. p < 0.05 was considered statistically significant and represented by a single *, whereas p < 0.005 is represented by **. Bars show mean ± SEM of three samples per timepoint. Note that the apparent increase in band intensities for some P5 samples (e.g. HDAC2, 3 and 4) reflect increased protein loading in those lanes.

To examine the temporal expression patterns and cellular localization of each HDAC in the proximal optic nerve, western blot and double-label immunohistochemistry were performed. The specificity of each antibody was tested initially by western blot analysis of proteins isolated from mouse optic nerve (Figure 5). Each antibody recognized a single band of the predicted molecular weight. The bands were then quantified by densitometry using β-TUBULIN as a loading control, and the levels of HDACs at each stage were graphed relative to E16 levels. Densitometric analysis indicated that the levels of a majority of HDACs were higher at E16, with falling levels at P5 and P30 (Figure 5). Protein levels showed temporal changes in expression corresponding to mRNA levels for most of the HDACs. Two exceptions to this observation were HDAC3 and 11. In the case of HDAC3, the mRNA decreased with developmental stage, whereas the protein levels increased. HDAC11 RNA increased substantially at P30, whereas the protein levels decreased. In addition, because RT-qPCR detected *Hdac7* mRNA in the optic nerve, we attempted to analyze HDAC7 protein expression in these studies. Unfortunately, none of the 5 antibodies tested were specific by western blot, precluding meaningful analysis of HDAC7 protein expression.

**Figure 6:**
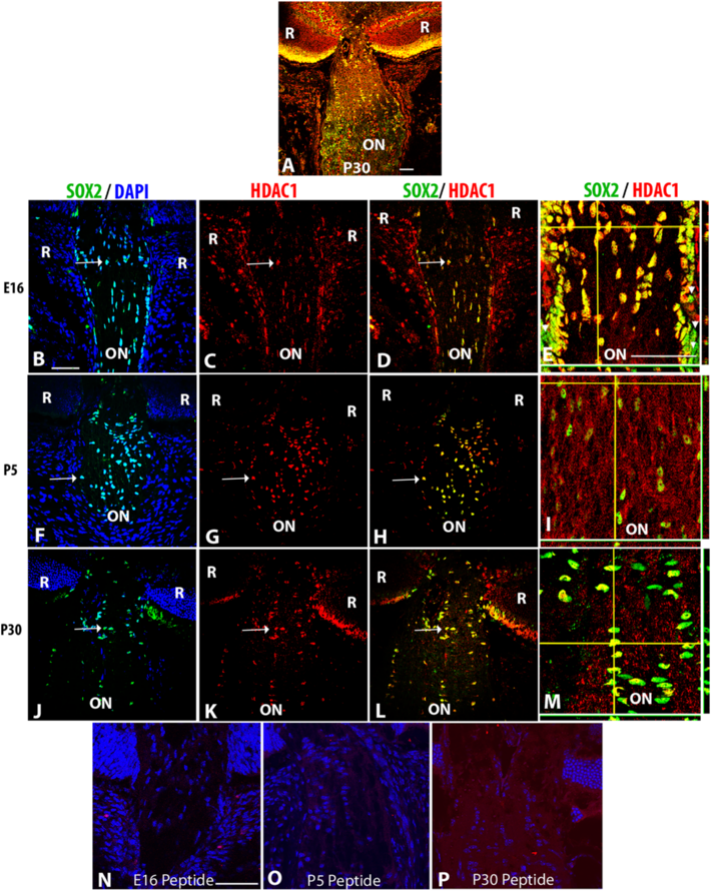
**HDAC1 localization pattern in developing murine optic nerve. (A)** Low magnification confocal micrograph showing the P30 optic nerve (ON), optic nerve head and retina (R) double-labeled for SOX2 (glia, green) and HDAC1 (red). Optic nerve sections at E16 **(B-E)**, P5 **(F-I)** and P30 **(J-M)** were triple-labeled with DAPI (blue), SOX2 (green) and HDAC1 (red). **(B, F, J)** Overlay of SOX2 and DAPI to aid in orientation of the image. Subsequent panels did not include DAPI to better show the co-localization of the SOX2 and HDAC labels. **(C, G, K)** HDAC1; **(D, H, L)** double-label of SOX2 and HDAC1. Arrows indicate nuclei that were co-labeled with SOX2 and HDAC1. Composite images in E, I and M show z stacks of optic nerve at high magnification; horizontal yellow lines correspond the x axis plane and vertical yellow lines corresponds to the y axis. The x, z axis is shown at the bottom of each panel, while the y, z axis is shown to the right of the panel. Negative controls at each stage showed absence of immunoreactivity following preabsorption of antibodies with the corresponding peptide immunogen **(N-P)**. Abbreviations: R; retina, ON; optic nerve. Scale bars = 50 μm. Scale bar in **B** applies to **B-D**, **F-H**, and **J-L**, scale bar in **E** applies to **E**, **I** and **M**.

**Figure 7:**
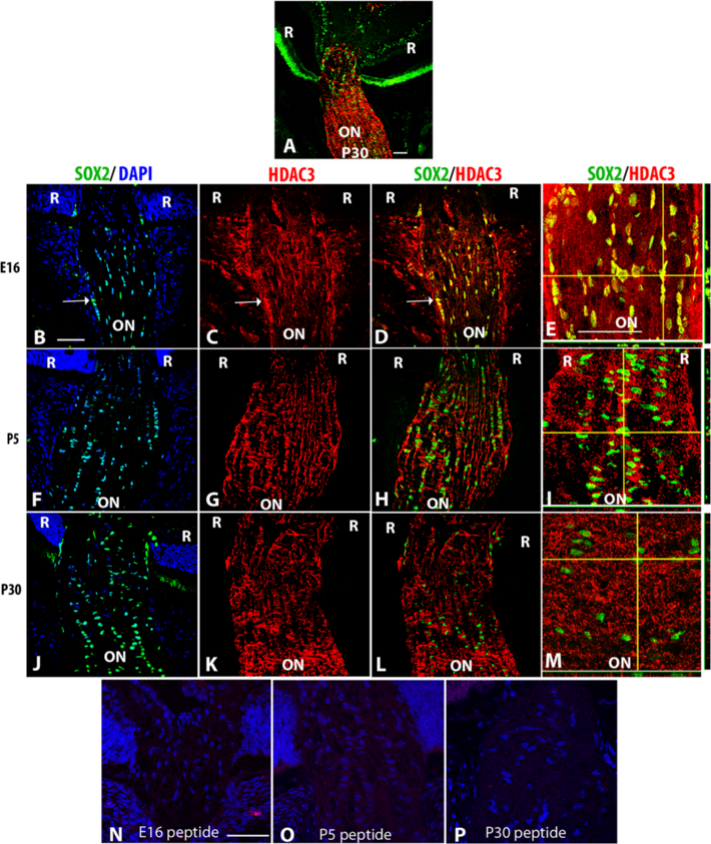
**HDAC3 localization pattern in developing murine optic nerve. (A)** Low magnification confocal micrograph showing the P30 optic nerve (ON), optic nerve head and retina (R) double-labeled for SOX2 (green) and HDAC3 (red). Optic nerve sections at E16 **(B-E)**, P5 **(F-I)** and P30 **(J-M)** were triple-labeled with DAPI (blue), SOX2 (green) and HDAC3 (red). **(B, F, J)** Overlays of SOX2 and DAPI to aid in orientation of the image. Subsequent panels did not include DAPI to better show the co-localization of the SOX2 and HDAC3 labels. **(C, G, K)** HDAC3; **(D, H, L)** double-label of SOX2 and HDAC3. Arrows indicate nuclei that were co-labeled with SOX2 and HDAC3. Composite images in E, I and M show z stacks of optic nerve at high magnification; horizontal yellow lines correspond the x axis plane and vertical yellow lines corresponds to the y axis. The x, z axis is shown at the bottom of each panel, while the y, z axis is shown to the right of the panel. Negative controls at each stage showed a lack of immunoreactivity following preabsorption of antibodies with the peptide immunogen **(N-P)**. Abbreviations: R; retina, ON; optic nerve. Scale bars = 50 μm. Scale bar in **B** applies to **B-D**, **F-H**, and **J-L**, and scale bar in **E** applies to **E**, **I** and **M**.

**Figure 8:**
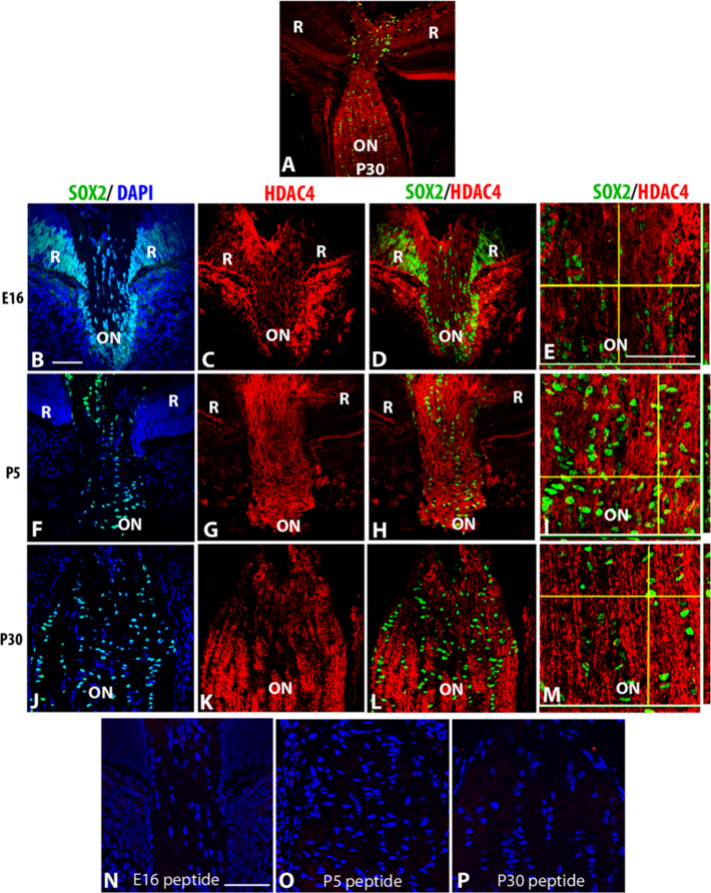
**HDAC4 localization pattern in developing murine optic nerve. (A)** Low magnification confocal micrograph showing the P30 optic nerve (ON), optic nerve head and retina (R) double-labeled for SOX2 (green) and HDAC4 (red). Optic nerve sections at E16 **(B-E)**, P5 **(F-I)** and P30 **(J-M)** were triple-labeled with DAPI (blue), SOX2 (green) and HDAC4 (red). **(B, F, J)**. Overlays of SOX2 and DAPI to aid in orientation of the image. Subsequent panels did not include DAPI to better show the co-localization of the SOX2 and HDAC3 labels. **(C, G, K)** HDAC4; **(D, H, L)** double-label of SOX2 and HDAC4. Composite images in **E**, **I** and **M** show z stacks of optic nerve at high magnification; horizontal yellow lines correspond the x axis plane and vertical yellow lines corresponds to the y axis. The x, z axis is shown at the bottom of each panel, while the y, z axis is shown to the right of the panel. Negative controls at each stage showed a lack of immunoreactivity following preabsorption of antibodies with the peptide immunogen **(N-P)**. Abbreviations: R; retina, ON; optic nerve. Scale bars = 50 μm. Scale bar in **B** applies to **B-D**, **F-H**, and **J-L**. Scale bar in **E** applies **E**, **I** and **M**.

To determine the cellular distribution of each HDAC in the developing optic nerve glia, sections of murine optic nerve and nerve head were double-labeled with SOX2 and antibodies specific for each HDAC. The analysis of HDAC immunoreactivity in the optic nerve revealed 3 basic patterns: 1) predominantly nuclear, 2) nuclear and cytoplasmic, or 3) cytoplasmic (see Table 1). Examples of each of the localization patterns are shown in Figures 6, 7, and 8, whereas the rest of the localization patterns are included as supplemental figures (Additional file 1: Figure S1, Additional file 2: Figure S2, Additional file 3: Figure S3, Additional file 4: Figure S4, Additional file 5: Figure S5, Additional file 6: Figure S6 and Additional file 7: Figure S7).

**Table 1:** Localization of HDACs in murine optic nerve

**Stages**	**Predominantly nuclear**	**Nuclear and cytoplasmic**	**Predominantly cytoplasmic**
E16	1, 2	3, 5	4, 6, 8, 9, 10, 11
P5	1, 2	5, 6, 8, 11	3, 4, 9, 10
P30	1, 2	5, 8	3, 4, 6, 9, 10, 11

HDACs 1 and 2 were predominantly nuclear in localization at all timepoints examined (Figure 6 and Additional file 1: Figure S1). In both cases, there was a high degree of overlap between SOX2+ nuclei and HDAC1 or HDAC2 immunoreactivity. However, at E16, there were also clearly SOX2+ cells at the periphery of the nerve that had little or no HDAC1 labeling (arrowheads, Figure 6E). There were also HDAC1 and HDAC2 + cells present within the optic nerve that were SOX2-negative. Since SOX2 is a marker of glial cells, HDAC+/SOX2-negative cells within the optic nerve itself were presumably endothelial cells or pericytes that are associated with blood vessels within the optic nerve.

HDACs 3, 5, 6, 8 and 11 were predominantly cytoplasmic, but showed nuclear co-labeling with SOX2 at one or more stages of development (Figure 7 and Additional file 1: Figure S2, Additional file 3: Figure S3 and Additional file 4: Figure S4 and Additional file 7: Figure S7). HDAC3 was co-localized with SOX2 in nearly all nuclei within the laminar region at E16, but only a few SOX2+ nuclei were also HDAC3+ at P5 and none of the SOX2+ nuclei were HDAC3+ at P30 (Figure 7). HDAC5 (Additional file 2: Figure S2) and HDAC 11 (Additional file 7: Figure S7) were rarely detected SOX2+ nuclei at P5 and those that were detected were located primarily near the outermost edge of the optic nerve. HDAC6 was co-localized with SOX2 in the laminar glia at P5 (Additional file 3: Figure S3), but was not detected in glia at other timepoints. HDAC8 (Additional file 4: Figure S4) was detected in a few SOX2+ nuclei located near the junction between the lamina and the more proximal regions of the optic nerve at P5 and P30. There was HDAC immunoreactivity in the optic nerve that was not localized to the nuclei and was therefore presumed to be cytoplasmic. Given the extensive non-nuclear staining for HDACs 3, 5, 6, 8 and 11, it is possible that some of the apparent co-localization was a result of overlap between their cytoplasmic staining and the nuclear SOX2 immunoreactivity. However, confocal imaging showed co-localization of SOX2 and the HDACs in both the x/z and y/z planes supporting at least some nuclear localization.

HDACs 4, 9, and 10 were predominantly cytoplasmic, with little to no nuclear label evident at any stage of development (Figure 8 and Additional file 5: Figure S5 and Additional file 6: Figure S6). For HDAC10, the apparent double labeling observed in low magnification image (Additional file 6: Figure S6) was an artifact that was not supported when examined at higher magnification using confocal imaging.

## Discussion

### Summary

To summarize the findings of these studies: 1) HDACs 1–11 were expressed in the optic nerve glial cells at all three stages of optic nerve development in the mouse, 2) HDACs 1 and 2 were predominantly nuclear throughout optic nerve development and glial cell maturation, 3) HDACs 3, 5, 6, 8,11 were predominantly cytoplasmic but showed nuclear localization in at least one stage of optic nerve development, 4) HDACs 4, 9 and10 were predominantly cytoplasmic, with little to no nuclear expression at any time during the developmental stages examined, and 5) nuclei co-labeled for AcH3K18 and MeH3K9 were present at similar levels throughout optic nerve development. In interpreting these data, we propose that both acetylation and deacetylation are required for astrocyte development within the optic nerve head and glial lamina. In light of the observation that HDACs 1, 2, 3, 5, 6, 8, and 11 were all co-localized to SOX2+ nuclei at some stages of development, these HDACs would be good candidates for further study to determine which interact with nuclear histones to regulate chromatin organization.

Our finding that HDAC2 is predominately nuclear in cells within the optic nerve differs from a recent report showing immunoreactivity for HDAC2 throughout the optic nerve in the nuclei of resident cells and the cytoplasm of the axons [36]. A major difference between the two studies is the ages of animals analyzed: in our developmental analysis, P30 was the latest age analyzed, whereas the other study analyzed expression in 12 month old mice. There were also regional differences in the portions of the optic nerve analyzed. In the present study, the area of the optic nerve head and glial lamina was analyzed, whereas in the previous report, the morphology of the nuclei would be more consistent with the non-laminar regions of the optic nerve closer to the optic chiasm. The non-laminar portions of the optic nerve contain the progeny of the oligodendrocyte precursors, whereas the laminar astrocytes are thought to derive from the intrinsic PAX2 expressing astrocyte precursors [5,29]. Therefore, the differences in HDAC2 expression could reflect the different lineages of the glial cells present in these distinct regions of the optic nerve. In addition, different antibodies were used, which could contribute to different patterns of immunoreactivity. It remains to be determined if these differences represent age-related or lineage-related changes in distribution of HDAC2. Interestingly, the patterns of HDAC1 and HDAC3 in the aging optic nerve appeared similar to that observed at P30 in our study, despite the differences in age, location within the optic nerve and in the primary antibodies used.

Many of the HDACs also appear to be localized to the cytoplasm in addition to or instead of the nucleus. The nuclear import of HDACs is blocked by post-translational modification or cleavage [37-44], therefore, the cytoplasmic localization of some HDACs reflects regulated protein trafficking between cellular compartments. In addition, HDACs have also been shown to deacetylate non-histone proteins resulting in changes in their structural and functional characteristics. For instance, HDAC6 interacts with cytoplasmic proteins such as tubulin and chaperones such as heat shock proteins (Hsp90), causing changes in the contractile functions of the muscles. Similarly, HDAC8 can deacetylate heat shock protein 20 (Hsp20) in the myometrium [45,46]. Hence the cytoplasmic localization of some HDACs may reflect an additional role for HDACs in deacetylation of cytoplasmic proteins during glial differentiation and maturation.

Protein levels showed temporal changes in expression that corresponded to mRNA levels for most of the HDACs consistent with general regulation at the transcriptional level. Two exceptions to this observation were HDACs 3 and 11, which showed increased mRNA levels at timepoints where protein levels were substantially decreased. Many factors can influence the conversion of mRNA into proteins including mRNA and/or protein half-life, post transcriptional regulation by micro RNAs [47,48]. HDACs are well-known targets of post-translational modifications that can affect their stability and activity, such as proteasome degradation via ubiquitinases or caspase mediated cleavage [49-52]. The E3 ubiquitin ligase SIAH2 interacts directly with HDAC3 and targets it for ubiquitin-dependent proteosomal degradation [53]. Although mechanisms regulating HDAC11 expression have not been characterized, there are reports of non-congruent changes in mRNA and protein levels in the murine brainstem and diencephalon at later stages of postnatal development [54]. Interestingly, in at least one study, HDAC4 mRNA was shown to have a much shorter half-life in comparison to the protein, leading differential increases in protein expression relative to mRNA levels [49]. Together, these findings are consistent with regulation of at least some *HDACs* at both transcriptional and post-transcriptional levels.

### Cells in the developing optic nerve do not appear to be globally acetylated or deacetylated

Several studies have concluded that the genome of differentiating neurons is globally acetylated [30,55], whereas other studies have proposed that acetylation is the key to the differentiation of oligodendrocytes [54,56-61]. In non-neuronal cells including glia, recruitment of HDACs by the RE-1 silencing transcription factor (REST) to the promoter of neuronal genes is critical to repress these genes and is thought to be essential for the development of glial cells [62-64]. Some of our data is consistent with the idea that deacetylation contributes to differentiation of astrocytes within the optic nerve head and laminar regions of the optic nerve; we found seven of the classic HDACs (1, 2, 3, 5, 6, 8, 11) are present in the nuclei of SOX2 positive glia in at least one or more stages during the astrocyte differentiation, consistent with a potential role in chromatin regulation. We did not examine the localization of HDAC7 due to lack of a sufficiently specific antibody, or expression of the sirtunins, the Class III HDACs. Therefore additional HDACs may be expressed in the glial precursors and differentiated cells that are not described in this study.

Many of the previous studies have focused on HDACs 1, 2, or 11 during differentiation of oligodendrocytes [54,59,65]. In contrast, very few studies have studied the role of epigenetics in astrocyte differentiation, but those that have been done appear to have conflicting results. Several studies using siRNA and morpholino antisense have shown that knocking down HDACs, in particular HDAC1 and 2, alters differentiation of both oligodendrocytes and neurons, without changing the number of astrocytes [30,31,61,66,67]. A region-specific role for HDACs has been identified in the telencephalon, inhibition of HDAC activity resulted in a BMP mediated decrease in neurogenesis and increase in astrogliogenesis by progenitors arising from the ganglionic eminences of the telencephalon *in vivo* and *in vitro*[68]. Further supporting a role in gliogenesis is the discovery that acetylation of histones associated with the STAT3 binding element in the GFAP promoter, a gene expressed typically in mature astrocytes, allows the LIF/STAT3 pathway to upregulate expression of GFAP [69]. Likewise, microarray analysis of neurosphere culture of forebrain cells treated with trichostatin A (to inhibit histone deacetylation) and BMP- (to promote astroglial differentiation) *in vitro* showed that STAT3 and phospho-STAT3, downstream members of the LIF and BMP pathways, were upregulated [70,71]. However, in the developing cortex in rats, neurogenesis was enhanced by HDAC inhibition *in vivo*, showing that there are regional and possibly lineage-specific differences in the functional contributions of HDAC activity to the neuronal/glial switch [68]. Some of the disparities in the studies of HDACs and astrocytes may be attributable to the fact that both *in vitro* and *in vivo* experiments are being compared. Epigenetic factors are regulated by cellular microenvironment and placing the cells *in vitro* has traditionally been a means of testing the effects of changing microenvironment on the cells. Therefore it should not come as surprise that epigenetic factors change when the cells are removed from the “normal” *in vivo* microenvironment.

The study presented here provides an excellent starting point for investigating the role of histone acetylation and deacetylation in optic nerve development and disease. Our data are consistent with contributions by not only HDAC1 and 2, but other HDACs that have not yet been actively studied. In addition to deacetylation, we also infer that acetylation is an intrinsic component to the differentiation of the laminar astrocytes. This is based on our finding that at all the three stages examined, antibodies specific for acetylated histone 3 immunolabeled increasing numbers of cells with increasing intensity across development. Whereas the studies herein are consistent with HDACs and HATs acting in combination to bring about macroglial differentiation, future studies will examine the roles of various HDACs and HATs at the genome level to ascertain their function at specific loci.

Interestingly, several studies have reported that systemic inhibition of HDAC activity can reduce RGC loss following injuries such as optic nerve crush [72,73], ischemia [36] and in the *Dba/2J* mouse model of pigment dispersion glaucoma [74]. However, an as yet unanswered question is how much of the rescue reflects direct effects on histone acetylation in retinal ganglion cells and how much is attributable to effects on the glial populations in the retina and/or optic nerve. Future studies will focus on testing the role of specific HDACs in glial cell development within the optic nerve and retina, as well as investigating potential changes in expression or activity levels in retinal diseases.

## Conclusion

The results of this study show that all the classical HDACs are present in the astrocytes during different stages of murine optic nerve development. Based on the localization pattern, the classical HDACs were placed into one of three different categories; predominantly nuclear, nuclear and cytoplasmic, and predominantly cytoplasmic. Seven of the classical HDACs, namely 1, 2, 3, 5, 6, 8, and 11, were localized to the nuclei of SOX2 positive glial cells in at least one or more stages of astrocyte differentiation in the murine optic nerve. HDACs 4, 9, and 10 were predominantly cytoplasmic throughout the development of the optic nerve astrocytes. Finally, HDAC7 was found in the optic nerve by RT-qPCR, but an antibody specific for HDAC7 was not available, hence the expression pattern of HDAC7 could not be determined. Understanding the localization pattern of HDACs during different stages of development of murine optic nerve provides a valuable resource to study the role of epigenetic regulation in the differentiation of the glial cell types, an area that has not been yet fully explored. Further, our data also provides an important avenue of future research, for understanding the potential role of epigenetic changes associated with disease processes such as glaucoma and diabetes.

## Methods

### Animal husbandry and tissue collection

Animals in this study were obtained and cared for in accordance with the Association for Research in Vision and Ophthalmology (ARVO) statement for the use of animals in ophthalmic and vision research and were housed in an AAALAC accredited housing facility in the School of Science on a 12 hour (hr) light/dark cycle. The ethical treatment of animals used for this study was approved by the IUPUI Science Animal Resource Center (protocol SC194R).

*C57BL/6J* mice were obtained from Jackson Labs (Bar Harbor, ME). For embryonic stages, mice were mated overnight and the females checked for vaginal plugs the next morning. The date the vaginal plug was detected was designated 0.5 days post coitum. At embryonic day 16 (E16), heads were removed and fixed in 4% paraformaldehyde in 0.1M phosphate buffer, pH 7.4 at 4°C for 24 hr. For postnatal stages, eyes were dissected and fixed in 4% paraformaldehyde in 0.1M phosphate buffer, pH7.4 at 4°C for 2 hrs. Following fixation, eyes were rinsed twice in 1X phosphate buffered saline (PBS; potassium chloride 200 mg/L, potassium phosphate 200 mg/L, sodium chloride 8000 mg/L, and sodium phosphate 1150 mg/L), pH 7.5 and placed in a graded series of sucrose in 0.1M phosphate buffer, starting at 5% and ending with 20%. Eyes were frozen in a 3:1 ratio of 20% sucrose in 0.1M phosphate buffer to Optimal Cutting Temperature (OCT; Sakura FineTek USA, Inc.) solution and stored at -80°C until sectioned.

### Immunohistochemistry

Ten micron sections were cut with a cryostat (Leica CM3050 S), placed on charged glass slides (Superfrost Plus; Fisher Scientific, Pittsburgh, PA), and stored at -80°C until used for immunohistochemistry. Slides were removed from the freezer, allowed to warm at room temperature for 30–40 minutes (min), liquid blocker applied to the regions surrounding the sections, and sections were post-fixed with 4% paraformaldehyde for 30 min at room temperature. Slides were washed in 1XPBS (2 × 2 min), incubated with methanol for 10 min at room temperature and washed twice with 1XPBS for (2 × 2 min). Antigen retrieval was performed by incubating sections in 1.0% SDS in 1XPBS for 5 min at room temperature. Slides were washed three times in 1XPBS (3 × 5 min) at room temperature, followed by treatment with 1.0% sodium borohydride for two min at room temperature to reduce autofluorescence. After removal of the sodium borohydride, sections were blocked with blocking solution (5% bovine serum albumin, 10% donkey serum, 0.25% triton X-100 in 1X Hanks balanced salt solution (HBSS) for 2 hrs at room temperature. Following removal of blocking solution, primary antibodies, diluted in 2% serum in 0.025% triton X, were added and incubated overnight at 4°C in a humid chamber. Dilutions and sources of each antibody are in Table 2.

**Table 2:** Concentration of antibodies used for IHC and Westerns

**Antibody**	**Source**	**Antibody dilution**		**Peptide supplier**
		**IHC**	**Westerns**	
Rabbit Anti-HDAC1 (Cat # ab33278)	ABCAM (Cambridge, MA)	1:300	1:2500	Abcam (Cambridge, MA)
Mouse Anti-HDAC2 (Cat # ab12169)	ABCAM (Cambridge, MA)	1:350	1:2500	Abcam (Cambridge, MA)
Rabbit Anti-HDAC3 (Cat # ab16047)	ABCAM (Cambridge, MA)	1:300	1:2500	Abcam (Cambridge, MA)
Mouse Anti-HDAC4 (Cat# NBP2–22151)	Novus Biologicals (Littleton, Co)	1:100	1:1500	Abcam (Cambridge, MA)
rabbit Anti-HDAC5 (Cat # ab53693)	ABCAM (Cambridge, MA)	1:100	1:750	Abcam (Cambridge, MA)
Goat Anti PAX2 (Cat # AF3364)	R and D Systems (Minneapolis, MN)	1:100	N/A	R and D Systems (Minneapolis, MN)
Rabbit Anti HDAC6 (Cat # LS-B5253)	Life Span Biosciences (Seattle, WA)	1:250	1:1000	Santa Cruz (Santa Cruz, CA)
Rabbit Anti-HDAC8 (Cat # LS-B967)	Life Span Biosciences (Seattle, WA)	1:500	1:750	Life Span Biosciences
Rabbit Anti-HDAC9 (Cat # ab59718)	ABCAM (Cambridge, MA)	1:250	1:500	Abcam (Cambridge, MA)
Rabbit Anti-HDAC10 (Cat # NB100-91801)	Novus Biologicals (Littleton, Co)	1:100	1:500	Santa Cruz (Santa Cruz, CA)
Rabbit Anti-HDAC11 (Cat # ab18973)	ABCAM (Cambridge, MA)	1:100	1:200	Abcam (Cambridge, MA))
Mouse Anti NKX2.2 (Cat # 74.5A5-s)	Developmental Hybridoma	1:10	N/A	N/A
Mouse Anti-methylated histone (cat # ab1220)	ABCAM (Cambridge, MA)	1:300	N/A	N/A
Rabbit Anti-Acetylated lysine (Cat # 9441)	Cell Signaling (Danvers, MA)	1:100	1:2000	N/A
Goat Anti-SOX2 (Cat # sc17320)	Santa Cruz (Santa Cruz, CA)	1:250	N/A	Santa Cruz (Santa Cruz, CA)
Mouse Anti SOX2 (Cat # ab75485	ABEAM (Cambridge, MA)	1:100	N/A	Abcam (Cambridge, MA))
Mouse anti-β-tubulin (Cat # T0198)	SIGMA (St. Louis, MO)	N/A	1:1000	N/A

Slides were then washed the next day in 1XPBS (2 × 10 min) at room temperature and were then incubated with secondary antibodies [Alexa-fluor conjugated secondaries (Donkey anti rabbit 549 and Donkey anti goat 488) from Invitrogen and DyLight conjugated secondaries (Donkey anti mouse 546) from Jackson ImmunoResearch] dissolved in 1XPBS at 1:800 dilution for 1 hr in dark at room temperature. Secondary antibodies were removed by washing the slides in 1XPBS (2 × 10 min). Sections used to label NKX2.2, the marker used for oligodendrocytes, were treated with biotinylated secondary antibody (BA9200, Vector laboratories) for 1 hr and then washed with 1XPBS (2X10 min). Incubation with Streptavidin 549 (SA-5549, Vector Laboratories) was then performed for 1 hr. Sections were mounted with anti-fade mounting medium containing DAPI (Prolong gold, Life Technologies). In some experiments, antibodies were preabsorbed with 32 μg/ml of the peptide or protein immunogen that was used to generate the antibodies for 1 hr prior to incubation with sections and immunofluorescence performed as usual. Additional sections were incubated with 0.25 μg/ml of IgG in blocking solution in place of the primary antibody and immunofluorescence performed as usual. Negative controls peptides corresponding to each HDAC are shown in Table 2. For all immunohistochemistry analysis, at least 3 independent samples were examined for antibody at each stage. Immunolabeling was imaged using an Olympus Fluoview FV 1000 confocal microscope. For Z stack images, an average of 22 to 25 optical sections were taken and merged together.

### Tissue analysis using cell counts

Confocal images of sections co labeled with SOX2 and PAX2, SOX2 and NKX2.2 were taken using FV1000 Olympus Fluoview microscope. Cells showing positive label in the optic nerve were counted over an area of 200 μm at all three stages (E16, P5 and P30) using retina on both sides as a reference. Cells were counted in every third section of through the optic nerve and three different nerves were analyzed for each timepoint.

### RT-qPCR

Optic nerves from the exit of the optic nerve up to, but not including the optic chiasm, were quickly isolated from mice following euthanasia and preserved in RNA stabilizing agent (RNAlater; Qiagen; Cat # 76104) at 4°C until ready for total RNA extraction. Total RNA was isolated using affinity columns (High Pure RNA Tissue Kit, Roche; Cat # 12033674001) with DNAse treatment. Quality analysis and quantification of total RNA was done using Nanodrop 2000c (ThermoScientific). For each RNA sample, cDNA was synthesized using oligo-dT primers as per manufacturer’s instructions (iScript cDNA Cat # 170–8891; Biorad). Briefly, cDNA was reverse transcribed by incubating 1μg of RNA template with nuclease-free water, 1X buffer reaction mix and reverse transcriptase at 25°C for 5 min, 42°C for 20 min, and 85°C for 5 min. cDNA was stored at -20°C until used for PCR reactions. Primer pairs (Table 3) were designed to detect all splice variants and spanned at least one intron. Specificity was verified using *in silico* PCR on the Santa Cruz Genome Browser [http://genome.ucsc.edu/], build mm10 [75]. Primer pairs for *Hdac1* also recognized a pseudogene located on chromosome X, but did not amplify in RT-minus reactions. Quantitative PCR reactions were processed in the investigator’s laboratory using Applied Biosystems Real Time PCR System (Model # 7300) in triplicate 20 μl reactions using SYBR green chemistry (Power SYBR Green Cat # 4368706; Applied Biosystems) according to manufacturer’s protocol with final primer concentration of 1μm and 4 μl of a 1:20 dilution of cDNA used in each reaction. Cycling conditions were: initial denaturation at 95°C for 10 min, 40 cycles of 95°C for 15 seconds (sec), 60°C for 30 sec, and 72°C for 30 sec, and a final extension of 72°C for 5 min. Melting curve analysis was performed with each PCR to verify single products were generated. Efficiency of each primer set was tested using the standard curve method: E = ((10^(-1/slope^)-1) X 100, where E = efficiency. Three reference genes, β-2 microglobulin (*B2m*), signal recognition particle 14 kDa (*Srp14*) and succinate dehydrogenase complex, subunit A (*Sdha*), were selected based on their reported consistent expression levels during rat retinal development [76] and in microarray data of gene expression during mouse eye and optic nerve development using the publicly available RefGene database [http://www.refgenes.org/rg/] [77]. Crossing thresholds (Ct) were determined by crossing point analysis (Additional file 8: Table S1). Data analysis and comparison of relative quantities used *Relative Expression Software Tool*-*Multiple Condition Solv*er (*REST-MCS*, [http://www.gene-quantification.de]) using the three reference genes for normalization [78]. This method uses a mathematical model that includes efficiency corrections and mean crossing-point deviation between the sample groups and control groups, with expression ratios tested for significance using a pair-wise fixed reallocation randomization test (2000 iterations); p ≤ 0.05 was considered statistically significant.

**Table 3:** List of primers used in RT-qPCR

**Gene**	**Accession number**	**Primer**	**Sequence (5′ to 3′)**	**Product length (bp)**	**Slope**	**SE (Slope)**	**Efficiency (%)**	**SE (Efficiency)**	**Pearson correlation**
***Hdac1***	NM_008228.2	Forward	TGGGGCTGGCAAAGGCAAGT	133	-3.586	±0.588	95.0%	±0.200	-0.996
		Reverse	GACCACTGCACTAGGCTGGAACA						
***Hdac2***	NM_ 008229.2	Forward	CGTACAGTCAAGGAGGCGGCAA	97	-3.361	±0.551	99.0%	±0.223	-0.996
		Reverse	TGAGGCTTCATGGGATGACCCTGG						
***Hdac3***	NM_010411.2	Forward	ACGTGCATCGTGCTCCAGTGT	150	-3.416	±0.42	98.0%	±0.163	-0.997
		Reverse	AGTGTAGCCACCACCTCCCAGT						
***Hdac4***	NM_207225.1	Forward	AGCTCTGGCAACGTCAGCACT	114	-3.347	±0.631	99.5%	±0.258	-0.993
		Reverse	AAGTGGGGCGACTGAGCCTTCT						
***Hdac5***	NM_001077696.1	Forward	ACGTAAATGTGGCGTGGACAGGA	147	-3.227	±0.264	102.0%	±0.119	-0.999
		Reverse	TTCAACAGCATCAAACCCAGCGGA						
***Hdac6***	NM_010413.3	Forward	TGGGGCTTCAAGGGCTGGATCT	148	-3.098	±0.380	105.0%	±0.192	-0.997
		Reverse	TGCTCTCTGATGGCATGGAGCC						
***Hdac7***	NM_001204275.1	Forward	GCTCAGCATGTGCATGTGGAACAC	132	-3.276	±0.520	101.0%	±0.226	-0.995
		Reverse	TGAGAGCCTGGTGTGTCTGGCT						
***Hdac8***	NM_027382.3	Forward	ATGACACCAGTGGTCGGCAA	105	-3.263	±0.656	101.5%	±0.288	-0.994
		Reverse	ACGAGCCGTGTTGGCAAGGTT						
***Hdac9***	NM_024124.3	Forward	TGCACCTTTGCCTCAGAGCACG	150	-3.373	±0.239	99.0%	±0.096	-0.999
		Reverse	TGGCTGCCTGGTTGCTTCAGT						
***Hdac10***	NM_199198.2	Forward	TAGCAGCCAAACATGCCAAGCAGA	143	-3.289	±0.329	100.5%	±0.0131	-0.998
		Reverse	ATGCTCATAGCGGTGCCAAGAGAAA						
***Hdac11***	NM_144919.2	Forward	GCTGGGAAATGGGGCAAGGTGA	134	-3.125	±0.728	104.5%	±0.358	-0.992
		Reverse	AGCTCGTTGAGATAGCGCCTCGT						
***Sdha***	NM_023281.1	Forward	GGACAGGCCACTCACTCTTAC	130	-3.493	±0.496	96.5%	±0.181	-0.997
		Reverse	CACAGTGCAATGACACCACG						
***Srp14***	NM_009273.4	Forward	TCGAGCCCGCAGAAAACA	145	-3.223	±0.373	102.0%	±0.169	-0.998
		Reverse	CTCTTCTTCAGCCCGTCCAT						
***β2-Microglobulin***	NM_009735.3	Forward	TCGCGGTCGCTTCAGTCGTC	135			105.1%		
		Reverse	CATTCTCCGGTGGGTGGCGTG						

### SDS-PAGE and western analysis

Isolated optic nerve tissue samples were lysed using lysis buffer (5M NaCl, 1M Tris pH 8.0, 0.5 M EDTA, 5% Triton-X 100 supplemented with protease inhibitor cocktail (Cat # P50700, RPI Corp) and 1 mM PMSF (Cat # 10837091001, Roche) on ice. Tissue lysates were centrifuged at 14000 rpm for 10 min at 4°C and supernatant was collected. Total protein concentration was determined using BCA protein Assay reagent (ThermoScientific). Proteins were resolved using SDS-PAGE (4-20% gradient gel), transferred to PVDF membranes, and blocked using 5% milk or protein-free tween 20 buffer (cat # 37571; ThermoScientific) and immunoblotted using specific HDAC antibodies. The concentrations of antibodies employed are specified in Table 2. The blots were stripped and re-probed for β-tubulin to check for equal protein loading. Densitometric analyses of the blots were performed using ImageJ 1.43u software Statistics was performed on the densitometric data using a one-way ANOVA with F-test and Scheffe procedure used for post hoc comparisons, with p < 0.05 considered statistically significant (IBM SPSS Statistics 20 software).

## Competing interests

The authors have no competing interests to declare in relation to this manuscript.

## Authors’ contributions

Experimental conception and design: TLB-A. Performance of experiments: ST, SD and MS. Analysis of results: TLB-A, ST and DCO. Manuscript preparation: TLB-A, DCO, ST, SD, and MS. All authors read and approved the final manuscript.

## Supplementary Material

Additional file 1: Figure S1 HDAC2 localization pattern in developing murine optic nerve. (A) Low magnification confocal micrograph showing the P30 optic nerve (ON), optic nerve head and retina (R) double-labeled for SOX2 (glia, (green) and HDAC2 (red). Optic nerve sections at E16 (B-E), P5 (F-I) and P30 (J-M) were triple-labeled with DAPI (blue), SOX2 (green) and HDAC2 (red). (B, F, J) Overlays of SOX2 and DAPI to aid in orientation of the image. Subsequent panels did not include DAPI to better show the co-localization of the SOX2 and HDAC2 labels. (C, G, K) HDAC2; (D, H, L) double-label of SOX2 and HDAC2. Arrows indicate nuclei that were co-labeled with SOX2 and HDAC2. Composite images in E, I and M show z stacks of optic nerve at high magnification; horizontal yellow lines correspond the x axis plane and vertical yellow lines corresponds to the y axis. The x,z axis is shown at the bottom of each panel, while the y,z axis is shown to the right of the panel . Negative controls at each stage showed a lack of immunoreactivity following preabsorption of antibodies with the peptide immunogen (N-P). Abbreviations: R; retina, ON; optic nerve. Scale bars = 50 μm. Scale bar in B applies to B-D, F-H, and J-L, scale bar in E applies to E, I and M. Click here for file

Additional file 2: Figure S2 HDAC5 localization pattern in developing murine optic nerve. (A) Low magnification confocal micrograph showing the P30 optic nerve (ON), optic nerve head and retina (R) double-labeled for SOX2 (glia, (green) and HDAC5 (red). Optic nerve sections at E16 (B-E), P5 (F-I) and P30 (J-M) were triple-labeled with DAPI (blue), SOX2 (green) and HDAC5 (red). (B, F, J) Overlays of SOX2 and DAPI to aid in orientation of the image. Subsequent panels did not include DAPI to better show the co-localization of the SOX2 and HDAC5 labels. (C, G, K) HDAC5; (D, H, L) double-label of SOX2 and HDAC5. Arrows indicate nuclei that were co-labeled with SOX2 and HDAC5. Composite images in E, I and M show z stacks of optic nerve at high magnification; horizontal yellow lines correspond the x axis plane and vertical yellow lines corresponds to the y axis. The x,z axis is shown at the bottom of each panel, while the y,z axis is shown to the right of the panel. Negative controls at each stage showed a lack of immunoreactivity following preabsorption of antibodies with the peptide immunogen (N-P). Abbreviations: R; retina, ON; optic nerve. Scale bars = 50 μm. Scale bar in B applies to B-D, F-H, and J-L, scale bar in E applies to E, I and M. Click here for file

Additional file 3: Figure S3 HDAC6 localization pattern in developing murine optic nerve*.* (A) Low magnification confocal micrograph showing the P30 optic nerve (ON), optic nerve head and retina (R) double-labeled for SOX2 (glia, (green) and HDAC6 (red). Optic nerve sections at E16 (B-E), P5 (F-I) and P30 (J-M) were triple-labeled with DAPI (blue), SOX2 (green) and HDAC6 (red). (B, F, J) Overlays of SOX2 and DAPI to aid in orientation of the image. Subsequent panels did not include DAPI to better show the co-localization of the SOX2 and HDAC6 labels. (C, G, K) HDAC6; (D, H, L) double-label of SOX2 and HDAC6. Arrows indicate nuclei that were co-labeled with SOX2 and HDAC6. Composite images in E, I and M show z stacks of optic nerve at high magnification; horizontal yellow lines correspond the x axis plane and vertical yellow lines corresponds to the y axis. The x,z axis is shown at the bottom of each panel, while the y,z axis is shown to the right of the panel . Negative controls at each stage showed a lack of immunoreactivity following preabsorption of antibodies with the peptide immunogen (N-P). Abbreviations: R; retina, ON; optic nerve. Scale bars = 50 μm. Scale bar in B applies to B-D, F-H, and J-L, scale bar in E applies to E, I and M. Click here for file

Additional file 4: Figure S4 HDAC8 localization pattern in developing murine optic nerve. (A) Low magnification confocal micrograph showing the P30 optic nerve (ON), optic nerve head and retina (R) double-labeled for SOX2 (glia, (green) and HDAC8 (red). Optic nerve sections at E16 (B-E), P5 (F-I) and P30 (J-M) were triple-labeled with DAPI (blue), SOX2 (green) and HDAC8 (red). (B, F, J) Overlays of SOX2 and DAPI to aid in orientation of the image. Subsequent panels did not include DAPI to better show the co-localization of the SOX2 and HDAC8 labels. (C, G, K) HDAC8; (D, H, L) double-label of SOX2 and HDAC8. Arrows indicate nuclei that were co-labeled with SOX2 and HDAC8. Composite images in E, I and M show z stacks of optic nerve at high magnification; horizontal yellow lines correspond the x axis plane and vertical yellow lines corresponds to the y axis. The x,z axis is shown at the bottom of each panel, while the y,z axis is shown to the right of the panel . Negative controls at each stage showed a lack of immunoreactivity following preabsorption of antibodies with the peptide immunogen (N-P). Abbreviations: R; retina, ON; optic nerve. Scale bars = 50 μm. Scale bar in B applies to B-D, F-H, and J-L, scale bar in E applies to E, I and M. Click here for file

Additional file 5: Figure S5 HDAC9 localization pattern in developing murine optic nerve*.* (A) Low magnification confocal micrograph showing the P30 optic nerve (ON), optic nerve head and retina (R) double-labeled for SOX2 (glia, (green) and HDAC9 (red). Optic nerve sections at E16 (B-E), P5 (F-I) and P30 (J-M) were triple-labeled with DAPI (blue), SOX2 (green) and HDAC9 (red). (B,F, J) Overlays of SOX2 and DAPI to aid in orientation of the image. Subsequent panels did not include DAPI to better show the co-localization of the SOX2 and HDAC9 labels. (C, G, K) HDAC9; (D, H, L) double-label of SOX2 and HDAC9. Composite images in E, I and M show z stacks of optic nerve at high magnification; horizontal yellow lines correspond the x axis plane and vertical yellow lines corresponds to the y axis. The x,z axis is shown at the bottom of each panel, while the y,z axis is shown to the right of the panel . Negative controls at each stage showed a lack of immunoreactivity following preabsorption of antibodies with the peptide immunogen (N-P). Abbreviations: R; retina, ON; optic nerve. Scale bars = 50 μm. Scale bar in B applies to B-D, F-H, and J-L, scale bar in E applies to E, I and M. Click here for file

Additional file 6: Figure S6 HDAC10 localization pattern in developing murine optic nerve*.* (A) Low magnification confocal micrograph showing the P30 optic nerve (ON), optic nerve head and retina (R) double-labeled for SOX2 (glia, (green) and HDAC10 (red). Optic nerve sections at E16 (B-E), P5 (F-I) and P30 (J-M) were triple-labeled with DAPI (blue), SOX2 (green) and HDAC10 (red). (B, F, J) Overlays of SOX2 and DAPI to aid in orientation of the image. Subsequent panels did not include DAPI to better show the co-localization of the SOX2 and HDAC10 labels. (C, G, K) HDAC10; (D, H, L) double-label of SOX2 and HDAC10. Composite images in E, I and M show z stacks of optic nerve at high magnification; horizontal yellow lines correspond the x axis plane and vertical yellow lines corresponds to the y axis. The x,z axis is shown at the bottom of each panel, while the y,z axis is shown to the right of the panel . Negative controls at each stage showed a lack of immunoreactivity following preabsorption of antibodies with the peptide immunogen (N-P). Abbreviations: R; retina, ON; optic nerve. Scale bars = 50 μm. Scale bar in B applies to B-D, F-H, and J-L, scale bar in E applies to E, I and M. Click here for file

Additional file 7: Figure S7 HDAC11 localization pattern in developing murine optic nerve*.* (A) Low magnification confocal micrograph showing the P30 optic nerve (ON), optic nerve head and retina (R) double-labeled for SOX2 (glia, (green) and HDAC11 (red). Optic nerve sections at E16 (B-E), P5 (F-I) and P30 (J-M) were triple-labeled with DAPI (blue), SOX2 (green) and HDAC11 (red). (B, F, J) Overlays of SOX2 and DAPI to aid in orientation of the image. Subsequent panels did not include DAPI to better show the co-localization of the SOX2 and HDAC11 labels. (C, G, K) HDAC11; (D, H, L) double-label of SOX2 and HDAC11. Arrows indicate nuclei that were co-labeled with SOX2 and HDAC11. Composite images in E, I and M show z stacks of optic nerve at high magnification; horizontal yellow lines correspond the x axis plane and vertical yellow lines corresponds to the y axis. The x,z axis is shown at the bottom of each panel, while the y,z axis is shown to the right of the panel . Negative controls at each stage showed a lack of immunoreactivity following preabsorption of antibodies with the peptide immunogen (N-P). Abbreviations: R; retina, ON; optic nerve. Scale bars = 50 μm. Scale bar in B applies to B-D, F-H, and J-L, scale bar in E applies to E, I and M. Click here for file

Additional file 8: Table S1 Crossing point analysis. Click here for file
