# Supplementary figures and images for: Histone deacetylase expression patterns in developing murine optic nerve
Source: BMC Dev Biol. 2014 Jul 9;14:30. doi: 10.1186/1471-213X-14-30 (PMC4099093; doi:10.1186/1471-213X-14-30)

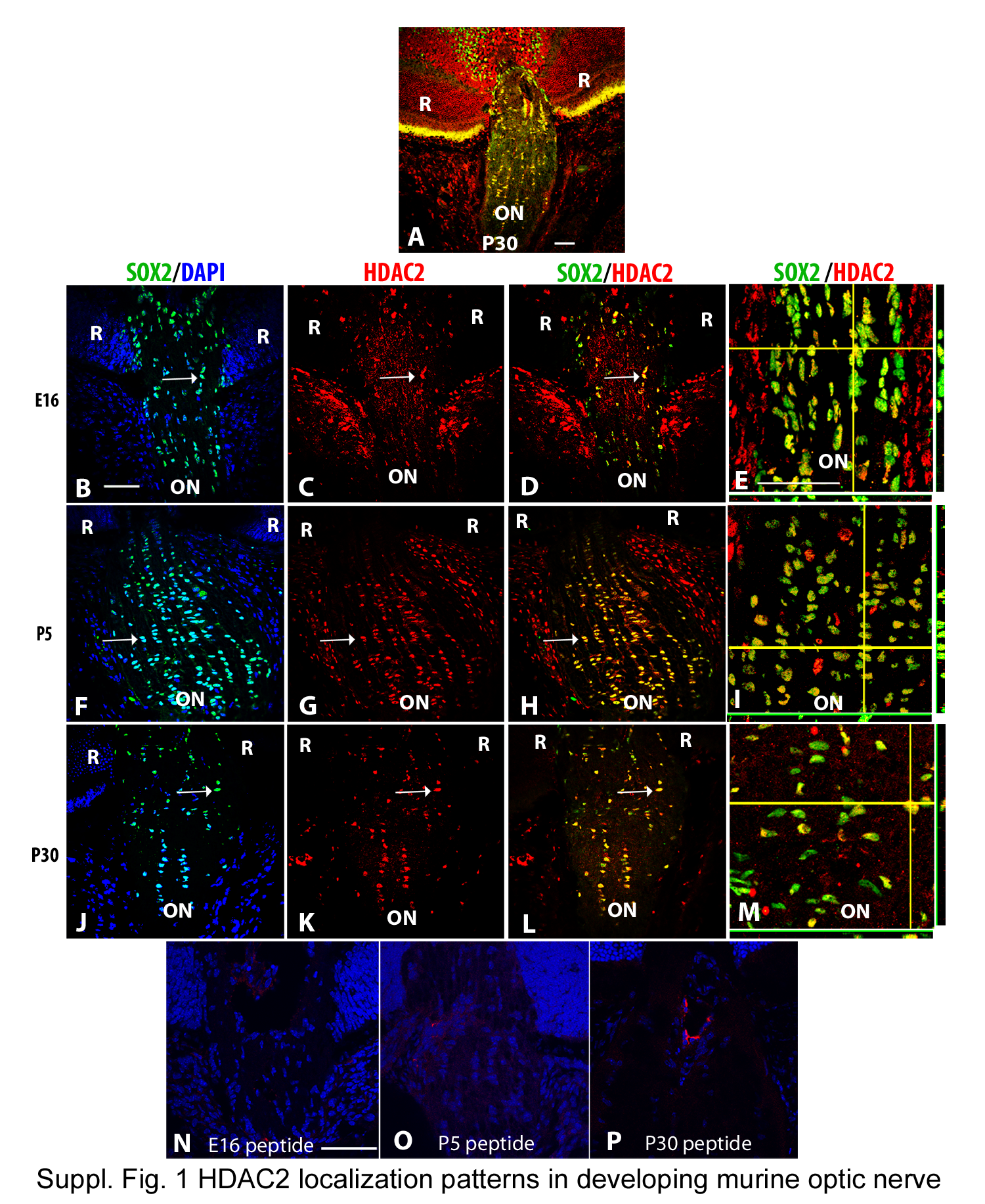

Supplement: Additional file 1: Figure S1 — HDAC2 localization pattern in developing murine optic nerve. (A) Low magnification confocal micrograph showing the P30 optic nerve (ON), optic nerve head and retina (R) double-labeled for SOX2 (glia, (green) and HDAC2 (red). Optic nerve sections at E16 (B-E), P5 (F-I) and P30 (J-M) were triple-labeled with DAPI (blue), SOX2 (green) and HDAC2 (red). (B, F, J) Overlays of SOX2 and DAPI to aid in orientation of the image. Subsequent panels did not include DAPI to better show the co-localization of the SOX2 and HDAC2 labels. (C, G, K) HDAC2; (D, H, L) double-label of SOX2 and HDAC2. Arrows indicate nuclei that were co-labeled with SOX2 and HDAC2. Composite images in E, I and M show z stacks of optic nerve at high magnification; horizontal yellow lines correspond the x axis plane and vertical yellow lines corresponds to the y axis. The x,z axis is shown at the bottom of each panel, while the y,z axis is shown to the right of the panel . Negative controls at each stage showed a lack of immunoreactivity following preabsorption of antibodies with the peptide immunogen (N-P). Abbreviations: R; retina, ON; optic nerve. Scale bars = 50 μm. Scale bar in B applies to B-D, F-H, and J-L, scale bar in E applies to E, I and M. [file 1471-213X-14-30-S1.png]

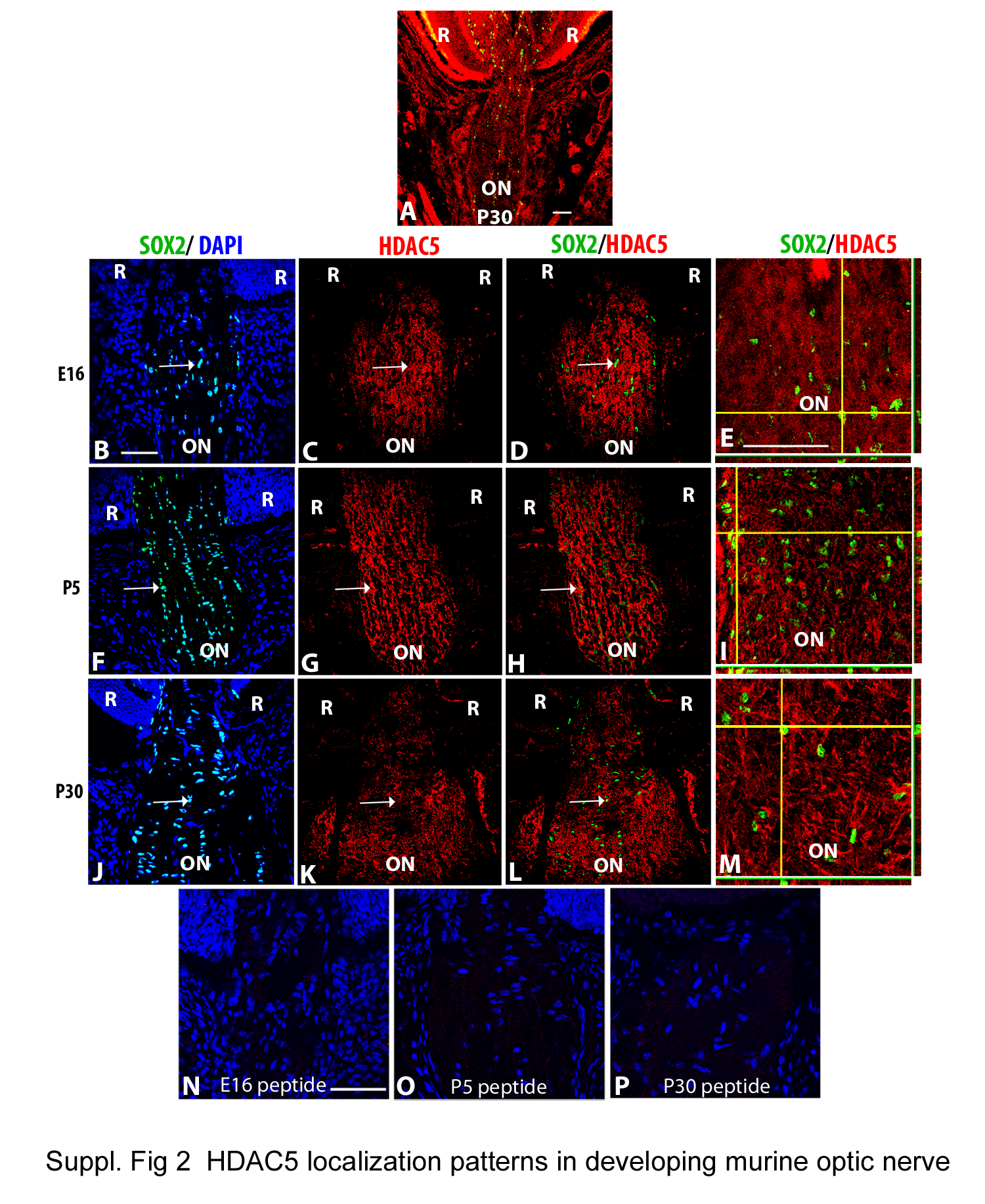

Supplement: Additional file 2: Figure S2 — HDAC5 localization pattern in developing murine optic nerve. (A) Low magnification confocal micrograph showing the P30 optic nerve (ON), optic nerve head and retina (R) double-labeled for SOX2 (glia, (green) and HDAC5 (red). Optic nerve sections at E16 (B-E), P5 (F-I) and P30 (J-M) were triple-labeled with DAPI (blue), SOX2 (green) and HDAC5 (red). (B, F, J) Overlays of SOX2 and DAPI to aid in orientation of the image. Subsequent panels did not include DAPI to better show the co-localization of the SOX2 and HDAC5 labels. (C, G, K) HDAC5; (D, H, L) double-label of SOX2 and HDAC5. Arrows indicate nuclei that were co-labeled with SOX2 and HDAC5. Composite images in E, I and M show z stacks of optic nerve at high magnification; horizontal yellow lines correspond the x axis plane and vertical yellow lines corresponds to the y axis. The x,z axis is shown at the bottom of each panel, while the y,z axis is shown to the right of the panel. Negative controls at each stage showed a lack of immunoreactivity following preabsorption of antibodies with the peptide immunogen (N-P). Abbreviations: R; retina, ON; optic nerve. Scale bars = 50 μm. Scale bar in B applies to B-D, F-H, and J-L, scale bar in E applies to E, I and M. [file 1471-213X-14-30-S2.png]

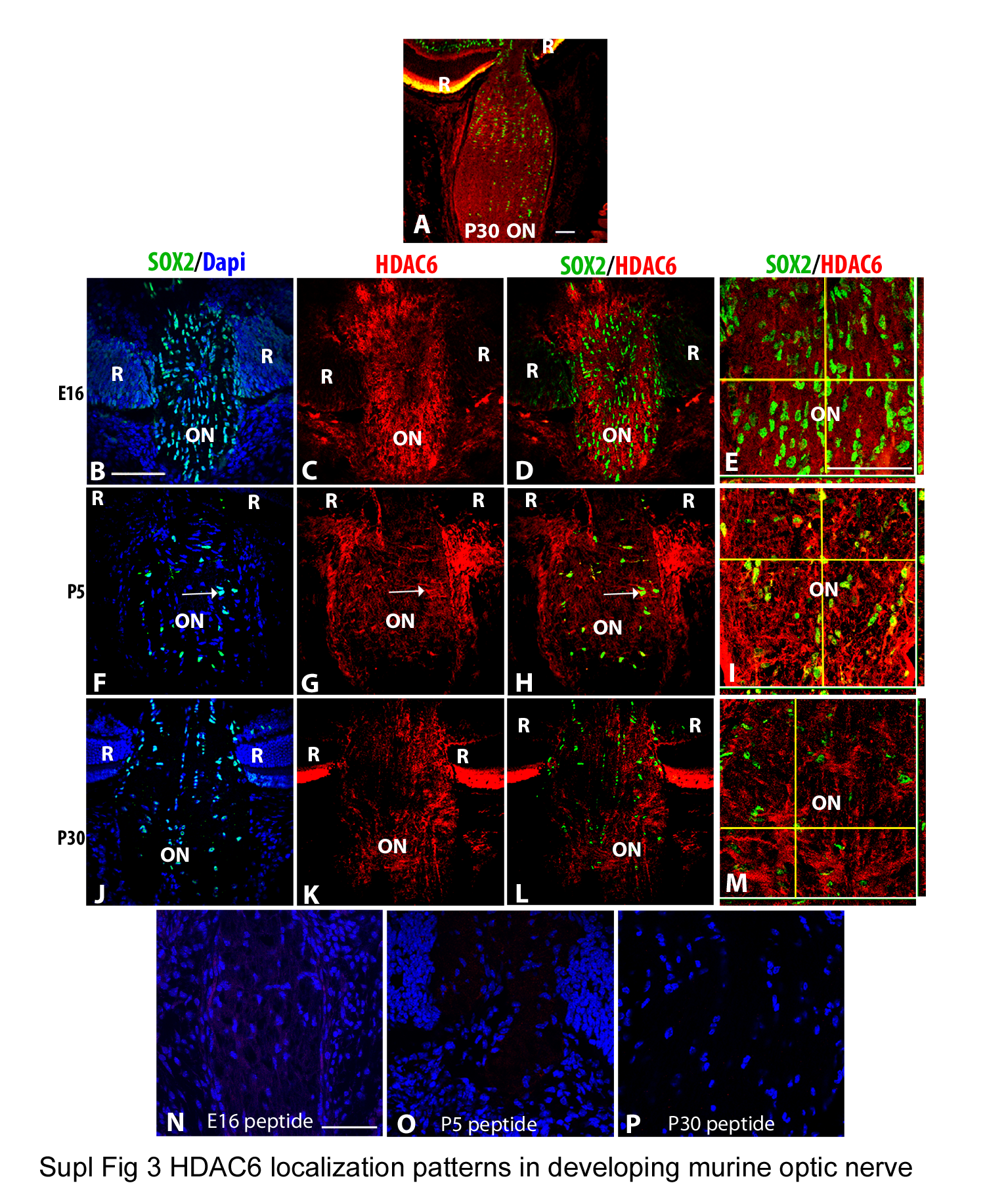

Supplement: Additional file 3: Figure S3 — HDAC6 localization pattern in developing murine optic nerve. (A) Low magnification confocal micrograph showing the P30 optic nerve (ON), optic nerve head and retina (R) double-labeled for SOX2 (glia, (green) and HDAC6 (red). Optic nerve sections at E16 (B-E), P5 (F-I) and P30 (J-M) were triple-labeled with DAPI (blue), SOX2 (green) and HDAC6 (red). (B, F, J) Overlays of SOX2 and DAPI to aid in orientation of the image. Subsequent panels did not include DAPI to better show the co-localization of the SOX2 and HDAC6 labels. (C, G, K) HDAC6; (D, H, L) double-label of SOX2 and HDAC6. Arrows indicate nuclei that were co-labeled with SOX2 and HDAC6. Composite images in E, I and M show z stacks of optic nerve at high magnification; horizontal yellow lines correspond the x axis plane and vertical yellow lines corresponds to the y axis. The x,z axis is shown at the bottom of each panel, while the y,z axis is shown to the right of the panel . Negative controls at each stage showed a lack of immunoreactivity following preabsorption of antibodies with the peptide immunogen (N-P). Abbreviations: R; retina, ON; optic nerve. Scale bars = 50 μm. Scale bar in B applies to B-D, F-H, and J-L, scale bar in E applies to E, I and M. [file 1471-213X-14-30-S3.png]

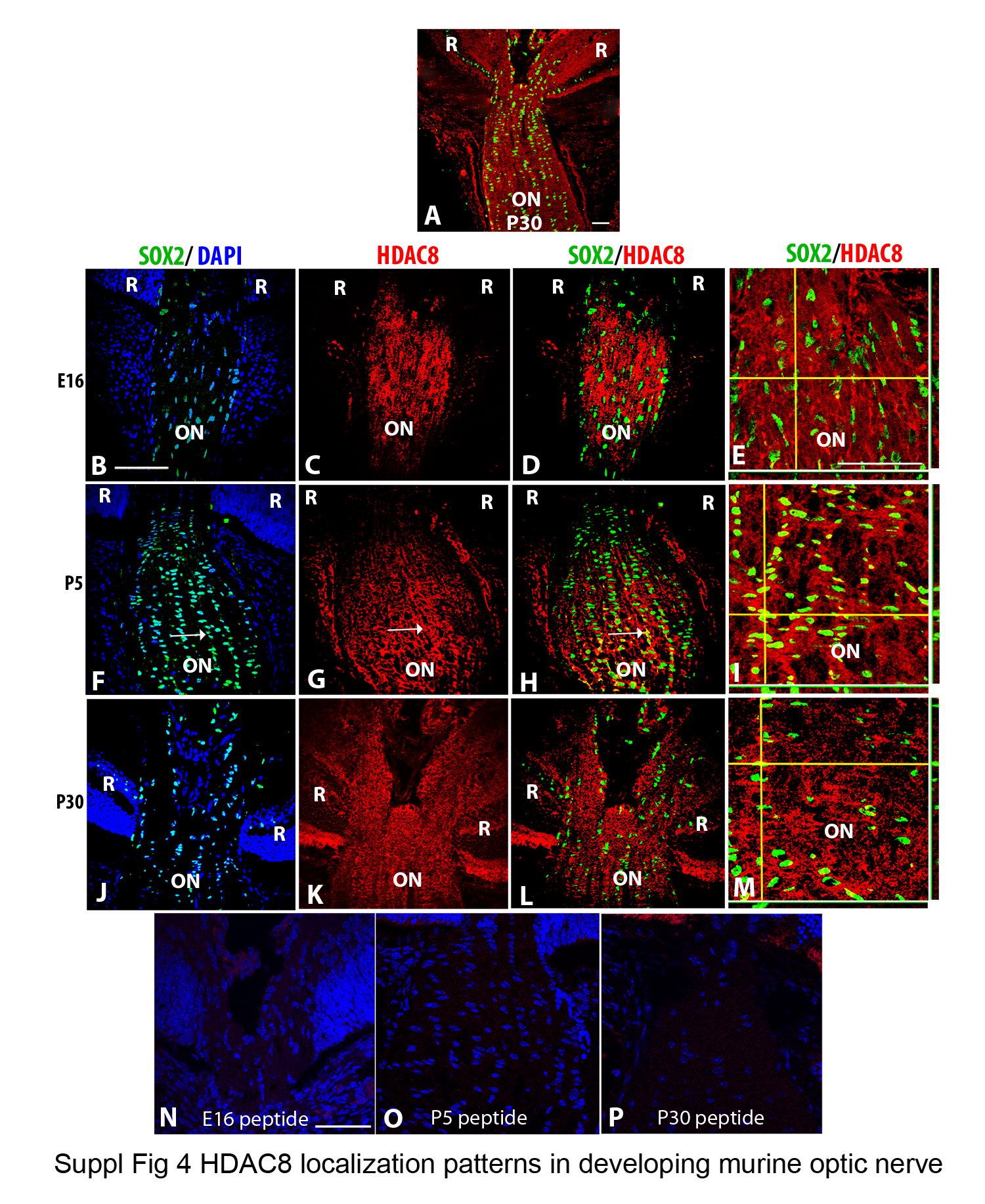

Supplement: Additional file 4: Figure S4 — HDAC8 localization pattern in developing murine optic nerve. (A) Low magnification confocal micrograph showing the P30 optic nerve (ON), optic nerve head and retina (R) double-labeled for SOX2 (glia, (green) and HDAC8 (red). Optic nerve sections at E16 (B-E), P5 (F-I) and P30 (J-M) were triple-labeled with DAPI (blue), SOX2 (green) and HDAC8 (red). (B, F, J) Overlays of SOX2 and DAPI to aid in orientation of the image. Subsequent panels did not include DAPI to better show the co-localization of the SOX2 and HDAC8 labels. (C, G, K) HDAC8; (D, H, L) double-label of SOX2 and HDAC8. Arrows indicate nuclei that were co-labeled with SOX2 and HDAC8. Composite images in E, I and M show z stacks of optic nerve at high magnification; horizontal yellow lines correspond the x axis plane and vertical yellow lines corresponds to the y axis. The x,z axis is shown at the bottom of each panel, while the y,z axis is shown to the right of the panel . Negative controls at each stage showed a lack of immunoreactivity following preabsorption of antibodies with the peptide immunogen (N-P). Abbreviations: R; retina, ON; optic nerve. Scale bars = 50 μm. Scale bar in B applies to B-D, F-H, and J-L, scale bar in E applies to E, I and M. [file 1471-213X-14-30-S4.png]

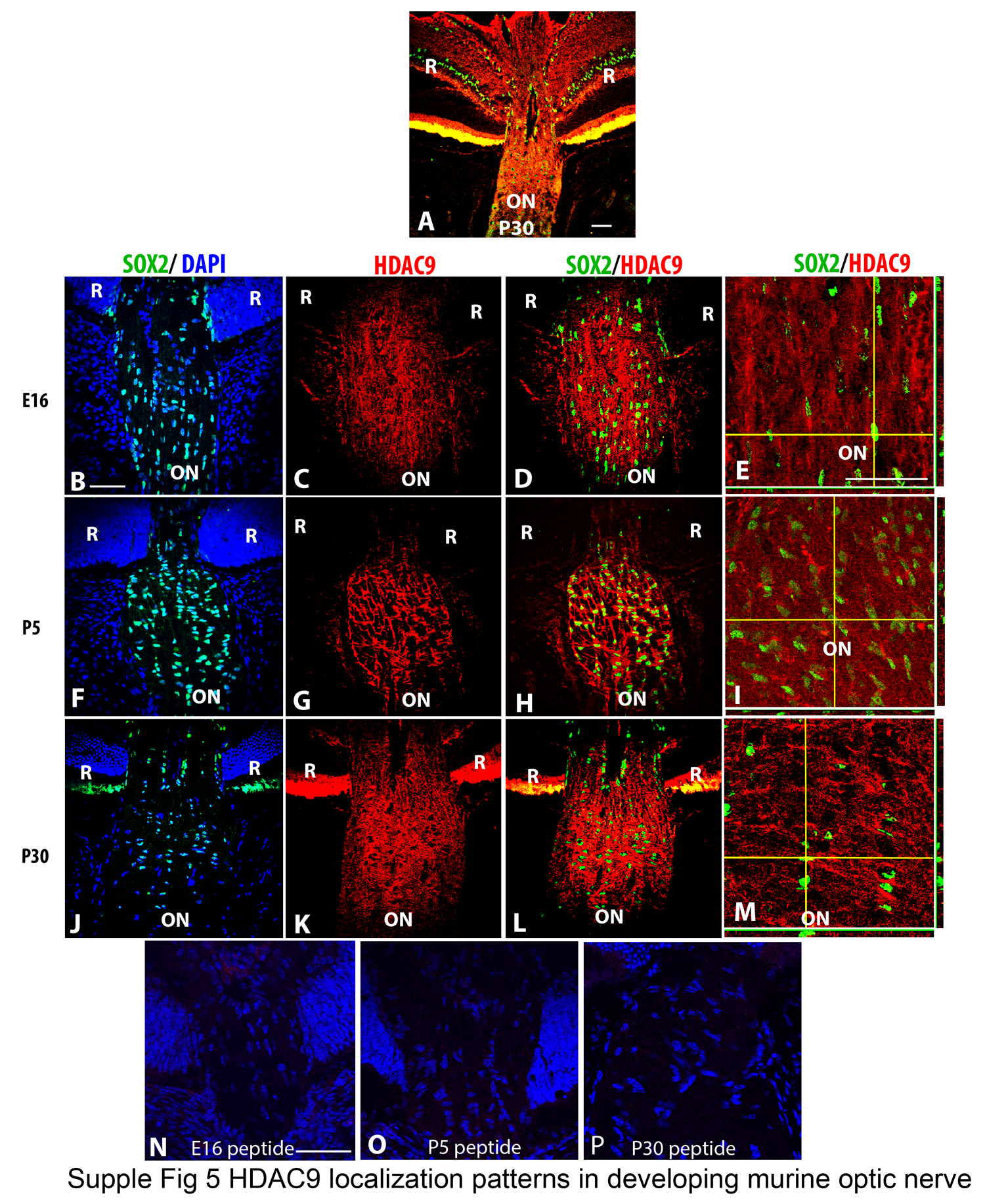

Supplement: Additional file 5: Figure S5 — HDAC9 localization pattern in developing murine optic nerve. (A) Low magnification confocal micrograph showing the P30 optic nerve (ON), optic nerve head and retina (R) double-labeled for SOX2 (glia, (green) and HDAC9 (red). Optic nerve sections at E16 (B-E), P5 (F-I) and P30 (J-M) were triple-labeled with DAPI (blue), SOX2 (green) and HDAC9 (red). (B,F, J) Overlays of SOX2 and DAPI to aid in orientation of the image. Subsequent panels did not include DAPI to better show the co-localization of the SOX2 and HDAC9 labels. (C, G, K) HDAC9; (D, H, L) double-label of SOX2 and HDAC9. Composite images in E, I and M show z stacks of optic nerve at high magnification; horizontal yellow lines correspond the x axis plane and vertical yellow lines corresponds to the y axis. The x,z axis is shown at the bottom of each panel, while the y,z axis is shown to the right of the panel . Negative controls at each stage showed a lack of immunoreactivity following preabsorption of antibodies with the peptide immunogen (N-P). Abbreviations: R; retina, ON; optic nerve. Scale bars = 50 μm. Scale bar in B applies to B-D, F-H, and J-L, scale bar in E applies to E, I and M. [file 1471-213X-14-30-S5.png]

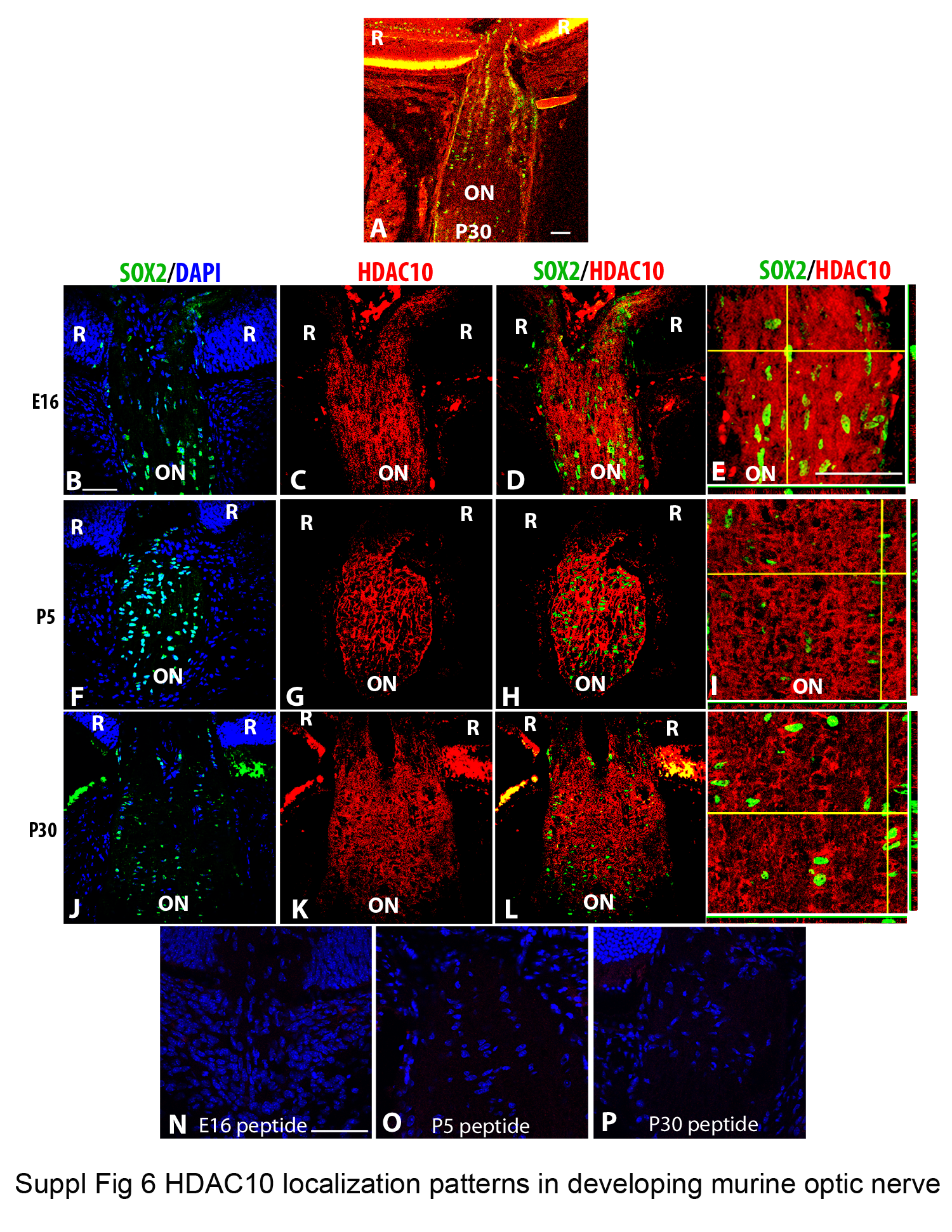

Supplement: Additional file 6: Figure S6 — HDAC10 localization pattern in developing murine optic nerve. (A) Low magnification confocal micrograph showing the P30 optic nerve (ON), optic nerve head and retina (R) double-labeled for SOX2 (glia, (green) and HDAC10 (red). Optic nerve sections at E16 (B-E), P5 (F-I) and P30 (J-M) were triple-labeled with DAPI (blue), SOX2 (green) and HDAC10 (red). (B, F, J) Overlays of SOX2 and DAPI to aid in orientation of the image. Subsequent panels did not include DAPI to better show the co-localization of the SOX2 and HDAC10 labels. (C, G, K) HDAC10; (D, H, L) double-label of SOX2 and HDAC10. Composite images in E, I and M show z stacks of optic nerve at high magnification; horizontal yellow lines correspond the x axis plane and vertical yellow lines corresponds to the y axis. The x,z axis is shown at the bottom of each panel, while the y,z axis is shown to the right of the panel . Negative controls at each stage showed a lack of immunoreactivity following preabsorption of antibodies with the peptide immunogen (N-P). Abbreviations: R; retina, ON; optic nerve. Scale bars = 50 μm. Scale bar in B applies to B-D, F-H, and J-L, scale bar in E applies to E, I and M. [file 1471-213X-14-30-S6.png]

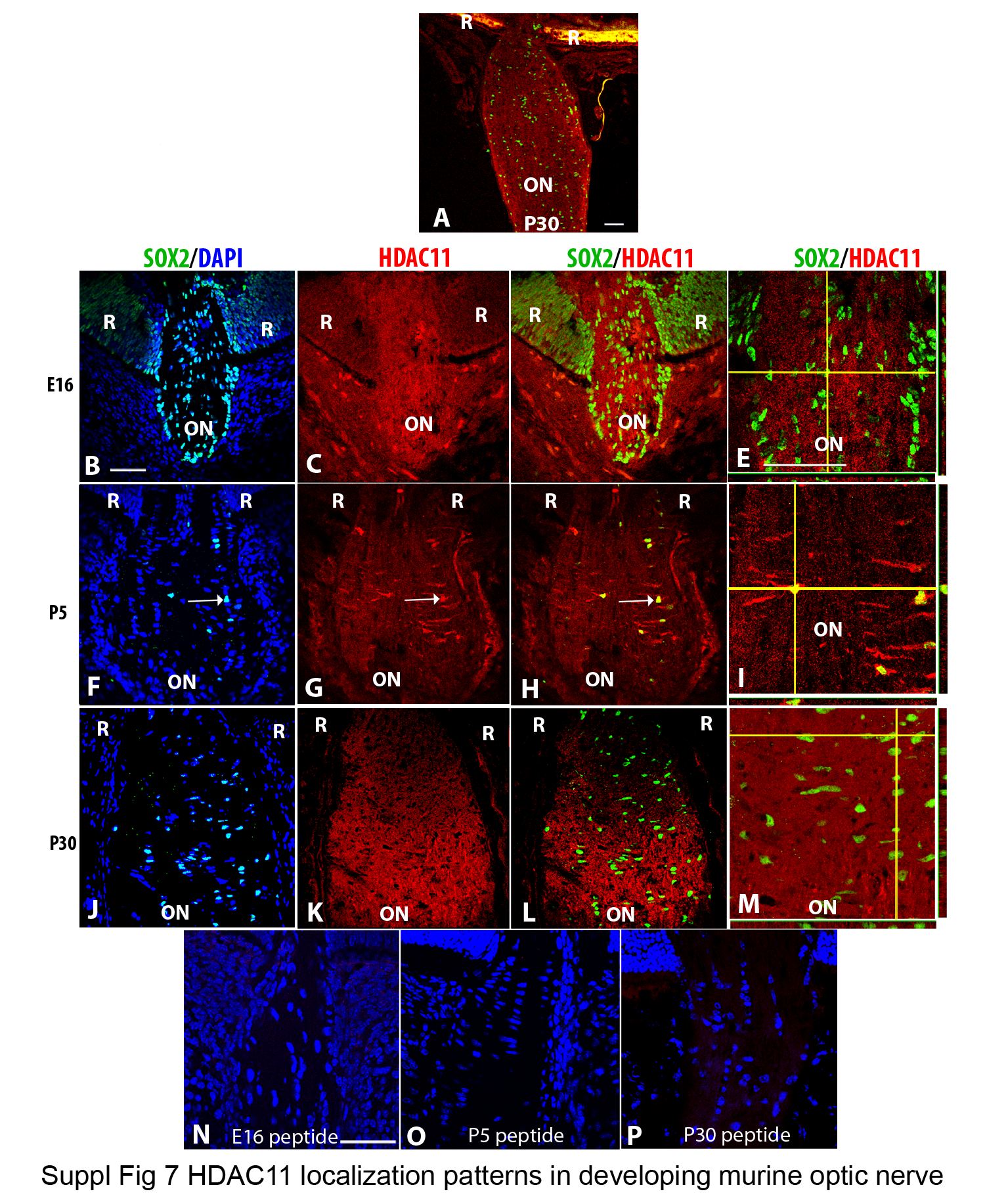

Supplement: Additional file 7: Figure S7 — HDAC11 localization pattern in developing murine optic nerve. (A) Low magnification confocal micrograph showing the P30 optic nerve (ON), optic nerve head and retina (R) double-labeled for SOX2 (glia, (green) and HDAC11 (red). Optic nerve sections at E16 (B-E), P5 (F-I) and P30 (J-M) were triple-labeled with DAPI (blue), SOX2 (green) and HDAC11 (red). (B, F, J) Overlays of SOX2 and DAPI to aid in orientation of the image. Subsequent panels did not include DAPI to better show the co-localization of the SOX2 and HDAC11 labels. (C, G, K) HDAC11; (D, H, L) double-label of SOX2 and HDAC11. Arrows indicate nuclei that were co-labeled with SOX2 and HDAC11. Composite images in E, I and M show z stacks of optic nerve at high magnification; horizontal yellow lines correspond the x axis plane and vertical yellow lines corresponds to the y axis. The x,z axis is shown at the bottom of each panel, while the y,z axis is shown to the right of the panel . Negative controls at each stage showed a lack of immunoreactivity following preabsorption of antibodies with the peptide immunogen (N-P). Abbreviations: R; retina, ON; optic nerve. Scale bars = 50 μm. Scale bar in B applies to B-D, F-H, and J-L, scale bar in E applies to E, I and M. [file 1471-213X-14-30-S7.png]
