# Supplementary material for: Histone deacetylase expression patterns in developing murine optic nerve
Source: BMC Dev Biol. 2014 Jul 9;14:30. doi: 10.1186/1471-213X-14-30 (PMC4099093; doi:10.1186/1471-213X-14-30)
Supplement: Additional file 8: Table S1 — Crossing point analysis. [file 1471-213X-14-30-S8.docx]

Supplemental Table I. Crossing threshold (Ct) value summary.

|  | E16 | | P5 | | P30 | |
| --- | --- | --- | --- | --- | --- | --- |
|  | Mean Ct | SEM | Mean Ct | SEM | Mean Ct | SEM |
| *Hdac1* | 28.82 | 0.48 | 30.24 | 1.02 | 28.87 | 0.58 |
| *Hdac2* | 26.17 | 0.47 | 28.58 | 0.49 | 27.86 | 0.24 |
| *Hdac3* | 29.73 | 0.40 | 28.29 | 0.61 | 27.82 | 0.64 |
| *Hdac4* | 32.38 | 0.51 | 31.12 | 0.42 | 28.85 | 0.37 |
| *Hdac5* | 32.74 | 0.14 | 31.07 | 0.34 | 32.03 | 0.43 |
| *Hdac6* | 29.65 | 0.34 | 33.36 | 0.29 | 30.54 | 0.56 |
| *Hdac7* | 31.99 | 0.30 | 29.48 | 0.16 | 31.79 | 0.29 |
| *Hdac8* | 33.67 | 0.33 | 30.76 | 0.10 | 29.93 | 0.62 |
| *Hdac9* | 32.26 | 0.41 | 30.46 | 0.05 | 29.83 | 0.36 |
| *Hdac10* | 28.62 | 1.03 | 29.72 | 0.03 | 29.95 | 0.16 |
| *Hdac11* | 31.95 | 0.32 | 34.41 | 0.02 | 30.06 | 0.48 |
|  | GeoMeanCt | SEM | GeoMeanCt | SEM | GeoMeanCt | SEM |
| Controls | 24.91 | 0.42 | 25.73 | 0.45 | 25.04 | 0.34 |

For experimental genes at each timepoint, the mean Ct was calculated across 9 reactions (3 biological replicates x 3 technical replicates). The standard error of the mean (SEM) was calculated based on 3 biological replicas. For controls, the geometric mean Ct (GeoMeanCt) was calculated for all 27 reactions (3 genes x 3 biological replicates x 3 technical replicates). The SEM was calculated based on 3 genes x 3 biological replicates.
